# Supplementary material for: How does the UK childcare energy-balance environment influence anthropometry of children aged 3–4 years? A cross-sectional exploration
Source: BMJ Open. 2018 Jul 12;8(7):e021520. doi: 10.1136/bmjopen-2018-021520 (PMC6082453; doi:10.1136/bmjopen-2018-021520)
Supplement: Supplementary file 1 [file bmjopen-2018-021520supp001.pdf]

Supplementary Table S1: Associations between childcare attendance and environment and BMI z-score (n=196)

	Exposure Measures β [95% CI]							
	Weekly hours in care		EPAO PA score		EPAO Nutrition score		EPAO total score	
	Unadjusted	Adjusted	Unadjusted	Adjusted	Unadjusted	Adjusted	Unadjusted	Adjusted
Constant	0.51 [0.22,0.81]	-0.35 [-1.27,0.56]	0.62 [-0.52,1.77]	-0.50 [-2.02,1.02]	0.36 [-0.82,1.53]	-0.45 [-1.91,1.00]	0.58 [-1.11,2.26]	-0.39 [-2.33,1.56]
**Child factors**								
Ethnicity (ref: white)								
White European		-0.06 [-0.56,0.44]		-0.10 [-0.61,0.40]		-0.10 [-0.61,0.40]		-0.10 [-0.61,0.41]
Other/ mixed		0.09 [-0.30,0.49]		0.05 [-0.35,0.45]		0.05 [-0.35,0.44]		0.05 [-0.34,0.45]
**Maternal Factors**								
Maternal BMI		0.05^{**} [0.02,0.08]		0.05^{***} [0.02,0.08]		0.05^{***} [0.02,0.08]		0.05^{***} [0.02,0.08]
Maternal education (ref: GCSE/ A-levels)								
Degree		-0.49^{**} [-0.83,-0.15]		-0.52^{**} [-0.86,-0.18]		-0.52^{**} [-0.86,-0.18]		-0.52^{**} [-0.86,-0.18]
Higher degree		0.04 [-0.31,0.39]		0.01 [-0.34,0.35]		0.01 [-0.33,0.36]		0.01 [-0.33,0.35]
Maternal working hours (ref: not employed)								
<20 hours		-0.37 [-0.79,0.04]		-0.40 [-0.81,0.02]		-0.40 [-0.81,0.02]		-0.40 [-0.81,0.02]
21-35 hours		-0.01 [-0.38,0.36]		-0.06 [-0.42,0.30]		-0.06 [-0.41,0.30]		-0.05 [-0.41,0.31]
>35 hours		0.20 [-0.21,0.61]		0.09 [-0.29,0.47]		0.09 [-0.29,0.47]		0.09 [-0.29,0.47]

95% confidence intervals in brackets; * $p < 0.05$, ** $p < 0.01$, *** $p < 0.001$

Supplementary Table S2: Associations between childcare attendance and environment and Waist to Height Ratio (n=188)

	Exposure Measures β [95% CI]							
	Weekly hours in care		EPAO PA score		EPAO Nutrition score		EPAO total score	
	Unadjusted	Adjusted	Unadjusted	Adjusted	Unadjusted	Adjusted	Unadjusted	Adjusted
Constant	0.48*** [0.36,0.60]	0.50*** [0.36,0.65]	0.42*** [0.25,0.59]	0.44*** [0.24,0.64]	0.51*** [0.34,0.67]	0.53*** [0.35,0.71]	0.44*** [0.23,0.65]	0.47*** [0.24,0.71]
**Child factors**								
Sex	0.01 [-0.02,0.03]	0.01 [-0.02,0.03]	0.01 [-0.02,0.03]	0.01 [-0.02,0.03]	0.01 [-0.02,0.03]	0.01 [-0.02,0.03]	0.01 [-0.02,0.03]	0.01 [-0.02,0.03]
Age in months	0.00 [-0.00,0.00]	0.00 [-0.00,0.00]	0.00 [-0.00,0.00]	0.00 [-0.00,0.00]	0.00 [-0.00,0.00]	0.00 [-0.00,0.00]	0.00 [-0.00,0.00]	0.00 [-0.00,0.00]
Ethnicity (ref: white)								
White European		0.01 [-0.04,0.06]		0.00 [-0.05,0.05]		0.01 [-0.04,0.05]		0.00 [-0.04,0.05]
Other/ mixed		0.00 [-0.04,0.04]		-0.00 [-0.04,0.04]		-0.00 [-0.04,0.04]		-0.00 [-0.04,0.04]
**Maternal Factors**								
Maternal BMI		-0.00 [-0.00,0.00]		-0.00 [-0.00,0.00]		-0.00 [-0.00,0.00]		-0.00 [-0.00,0.00]
Maternal education (ref: GCSE/ A-levels)								
Degree		0.00 [-0.03,0.03]		0.00 [-0.03,0.03]		-0.00 [-0.03,0.03]		0.00 [-0.03,0.03]
Higher degree		-0.00 [-0.03,0.03]		-0.00 [-0.03,0.03]		-0.00 [-0.04,0.03]		-0.00 [-0.04,0.03]
Maternal working hours (ref: not employed)								
<20 hours		0.01 [-0.03,0.05]		0.00 [-0.04,0.04]		0.00 [-0.04,0.04]		0.00 [-0.04,0.04]
21-35 hours		0.01 [-0.02,0.05]		0.01 [-0.03,0.04]		0.01 [-0.03,0.04]		0.01 [-0.03,0.04]
> 35 hours		-0.00 [-0.04,0.03]		-0.01 [-0.05,0.02]		-0.01 [-0.05,0.02]		-0.01 [-0.05,0.02]

95% confidence intervals in brackets; * $p < 0.05$, ** $p < 0.01$, *** $p < 0.001$

Supplementary Table S3: Associations between childcare attendance and environment and sum of skinfolds thickness (n=144)

	Exposure Measures β [95% CI]							
	Weekly hours in care		EPAO PA score		EPAO Nutrition score		EPAO total score	
	Unadjusted	Adjusted	Unadjusted	Adjusted	Unadjusted	Adjusted	Unadjusted	Adjusted
Constant	15.12*** [9.80,20.44]	11.66*** [6.03,17.30]	15.71*** [8.20,23.23]	10.17* [2.07,18.27]	16.38*** [9.12,23.64]	14.30*** [6.81,21.78]	15.96*** [6.75,25.17]	12.09* [2.43,21.76]
**Child factors**								
Sex	1.18* [0.18,2.19]	1.41** [0.47,2.35]	1.25* [0.23,2.27]	1.53** [0.57,2.49]	1.24* [0.22,2.26]	1.52** [0.57,2.48]	1.25* [0.23,2.27]	1.53** [0.57,2.49]
Age in months	-0.02 [-0.13,0.09]	-0.05 [-0.16,0.05]	-0.03 [-0.14,0.08]	-0.06 [-0.17,0.05]	-0.03 [-0.14,0.08]	-0.06 [-0.17,0.05]	-0.03 [-0.14,0.08]	-0.06 [-0.17,0.05]
Ethnicity (ref: white)								
White European		-0.18 [-1.94,1.58]		-0.35 [-2.17,1.48]		-0.20 [-2.00,1.61]		-0.25 [-2.07,1.58]
Other/ mixed		-0.04 [-1.51,1.42]		-0.01 [-1.51,1.50]		0.08 [-1.40,1.55]		0.10 [-1.39,1.59]
**Maternal Factors**								
Maternal BMI		0.27*** [0.15,0.40]		0.27*** [0.15,0.40]		0.27*** [0.15,0.40]		0.27*** [0.14,0.40]
Maternal education (ref: GCSE/ A-levels)								
Degree		-1.61** [-2.81,-0.41]		-1.46* [-2.67,-0.24]		-1.49* [-2.70,-0.28]		-1.49* [-2.71,-0.28]
Higher degree		-0.27 [-1.54,1.00]		0.13 [-1.13,1.40]		0.16 [-1.11,1.44]		0.09 [-1.18,1.35]
Maternal working hours (ref: not employed)								
<20 hours		-1.03 [-2.44,0.37]		-0.93 [-2.36,0.50]		-0.93 [-2.36,0.50]		-0.90 [-2.33,0.53]
21-35 hours		-0.30 [-1.64,1.04]		-0.01 [-1.34,1.31]		0.07 [-1.24,1.39]		0.04 [-1.29,1.37]
>35 hours		-0.36 [-1.82,1.09]		-0.05 [-1.42,1.32]		-0.04 [-1.41,1.33]		-0.05 [-1.42,1.33]

95% confidence intervals in brackets; * $p < 0.05$, ** $p < 0.01$, *** $p < 0.001$
