# Supplementary material for: How does the UK childcare energy-balance environment influence anthropometry of children aged 3–4 years? A cross-sectional exploration
Source: BMJ Open. 2018 Jul 12;8(7):e021520. doi: 10.1136/bmjopen-2018-021520 (PMC6082453; doi:10.1136/bmjopen-2018-021520)
Supplement: Author's manuscript [file bmjopen-2018-021520.draft_revisions.pdf]

BMJ Open

BMJ Open is committed to open peer review. As part of this commitment we make the peer review history of every article we publish publicly available.

When an article is published we post the peer reviewers' comments and the authors' responses online. We also post the versions of the paper that were used during peer review. These are the versions that the peer review comments apply to.

The versions of the paper that follow are the versions that were submitted during the peer review process. They are not the versions of record or the final published versions. They should not be cited or distributed as the published version of this manuscript.

BMJ Open is an open access journal and the full, final, typeset and author-corrected version of record of the manuscript is available on our site with no access controls, subscription charges or pay-per-view fees (http://bmjopen.bmj.com).

If you have any questions on BMJ Open's open peer review process please email info.bmjopen@bmj.com

BMJ Open

The childcare energy-balance environment and UK 3-4-year-olds' anthropometry

Journal:	*BMJ Open*
Manuscript ID	bmjopen-2018-021520
Article Type:	Research
Date Submitted by the Author:	08-Jan-2018

Complete List of Authors:	Hesketh, Kathryn R.; UCL Institute of Child Health, Population, Policy and Practice; University of Cambridge, CEDAR and MRC Epidemiology Unit<br>Benjamin Neelon, Sara E.; Johns Hopkins University, Department of Health, Behavior and Society, Bloomberg School of Public Health<br>van Sluijs, Esther; University of Cambridge, CEDAR and MRC Epidemiology Unit

Keywords:	Childcare environment, anthropometry, Preschool, Nutrition < TROPICAL MEDICINE, physical activity

SCHOLARONE™
Manuscripts

The childcare energy-balance environment and UK 3-4-year-olds' anthropometry

Authors

Kathryn R. Hesketh[1,2*]

Sara E. Benjamin Neelon[1,2]

Esther M.F. van Sluijs[1]

[1] CEDAR and MRC Epidemiology Unit, University of Cambridge, Cambridge, UK

[2] Department of Health, Behavior and Society, Johns Hopkins Bloomberg School of Public Health, Baltimore, MD, USA

[*] Corresponding author:

Kathryn.hesketh@ucl.ac.uk

krh40@cam.ac.uk

Address: CEDAR and MRC Epidemiology Unit, University of Cambridge School of Clinical Medicine, Cambridge, UK

Telephone: 01223 769169

Word count: 3873

Figures: 0

Tables: 3

Key words: Childcare environment; physical activity; nutrition; Preschool; anthropometry

For peer review only

For peer review only - http://bmjopen.bmj.com/site/about/guidelines.xhtml

ABSTRACT

Objectives To assess the association between time spent in care, the childcare energy-balance environment, and preschoolers' z-BMI, waist-to-height ratio (WHR) and sum of skinfolds thickness (SST).

Design Cross-sectional study.

Setting and participants 3-4-year-old children were recruited from 30 childcare Cambridgeshire centres (UK) in 2013.

Main outcome measures Objectively measured height and weight was used to calculate BMI z-score; waist circumference and height were used to generate WHR; subscapular and tricep skinfolds were used to calculate SST. Associations between childcare attendance/environment and outcomes were explored using two-level hierarchical regression models, adjusting for demographic and family-based confounders.

Results 196 children (49% female) provided valid data. Time spent in care, and the nutrition, physical activity, and overall childcare environment were not associated with children's z-BMI score, WHR and SST.

Conclusions Childcare environment and level of attendance were not associated with UK preschool-aged children's anthropometry. The childcare environment has been central to intervention efforts to prevent/ reduce early childhood obesity, yet family-level factors warrant substantial attention when considering obesity prevention strategies for young children.

STRENGTHS AND LIMITATIONS OF THIS STUDY

- We used objective measures of UK preschoolers' anthropometric indices to provide novel information about how they are associated with childcare attendance and environment.

For peer review only - http://bmjopen.bmj.com/site/about/guidelines.xhtml

- This was a relatively small UK childcare-based sample, but our outcomes and exposures were normally distributed, providing sufficient heterogeneity to explore our research questions.
- We used hierarchical regression analysis to take account of study design and potential clustering at the centre-level.
- We also adjusted for a number of family-level factors known to be related to children's anthropometry
- We did not have a measure of each child's birth-weight to account for children's individual growth trajectories, nor previous childcare attendance to assess prospective associations.

INTRODUCTION

In 2010, 43 million preschool-aged children worldwide were estimated to be overweight or obese, with a further 92 million at risk of overweight/obesity.[1] Although levels appear to be stabilizing,[2] obesity in childhood is associated with a range of unfavourable outcomes including type 2 diabetes, hyperlipidaemia, and psychosocial problems.[3]

Obesity is often described as an imbalance between energy intake (food consumption) and energy expenditure (physical activity) resulting in excess weight gain over time.[4] As early childhood is a period of rapid growth and development, it represents a key time for establishing healthy energy balance related behaviours (EBRBs), which include physical activity, sedentary and dietary behaviours. Whilst parents provide the majority of care for children before they enter school, children now spend increasing amounts of time in out-of-home care prior to starting formal education.[5]

In 2014, the average enrolment rate of 3-5 year-olds in (pre-school) educational programmes

For peer review only - http://bmjopen.bmj.com/site/about/guidelines.xhtml

in OECD countries was 80%.[5] Consequently, preschool-aged children, and particularly those who spend large amounts of time in formal care each week, rely on childcare centres to provide a significant proportion of their food and physical activity opportunities. Elements such as staff education and training, staff behaviour on the playground, lower playground density (less children per square meter), the presence of vegetation and portable toys, and open play areas appear to be positively related to children's physical activity.[6] Likewise, childcare centres and specifically, individual childcare provider practices, appear to be associated with positive dietary behaviours in young children.[7]

Given the influence of the childcare environment on children's EBRBs, and the importance of EBRBs for children maintaining a healthy weight, it follows that time spent in childcare is likely to influence children's weight status. Indeed a number of studies assessing children during infancy and preschool suggest that those children attending formal care (vs. those not in formal care) are more likely to be overweight/ obese,[8,9] with both nutrition and physical activity environments contributing.[10] In contrast, a recent systematic reviews assessing the link between childcare attendance and obesity-related outcomes found inconsistent results. Black and colleagues assessed 24 studies (17 of which were longitudinal) conducted in North America (n=15), Europe (n=7) and Asia (n=2). In thirteen studies, there was an increased risk of overweight/obesity for children attending informal (n = 9) or centre-based care (n = 4) compared to parental care; in seven studies a decreased risk of overweight/obesity in centre vs. informal/parental care; and four studies reported no association between any form of care and overweight/obesity.[11] There is also some uncertainty as to whether this risk differs by socio-demographic factors: children from lower socio-economic backgrounds who spend time in childcare have been shown to have both a greater[12] and lower[13] risk of developing obesity.

For peer review only - http://bmjopen.bmj.com/site/about/guidelines.xhtml

Due to the high numbers of children attending formal out-of-home care, and the reach and ease of intervening in these environments, intervention studies are often developed in these settings to prevent, halt or reverse obesity during the preschool years. A review by Zhou and colleagues[14] identified 15 such studies: six (of 13) interventions with a dietary component, and eight (of 12) interventions with physical activity component reported improvements in the target EBRB,[14] with seven (47%) reporting subsequent improvements in children's adiposity. Few studies (4/15) assessed long-term efficacy or sustainability (5/15) and the authors also noted heterogeneity across study designs and the interventions delivered, which suggests effects may be limited to specific population subgroups.

Such findings perhaps reflect differences in policies and practices around nutrition and physical activity across childcare centres (e.g. by level of deprivation[15]), and between countries that are likely to influence the extent to which the childcare environment is associated with children's weight indicators. Moreover, uptake of childcare varies between countries,[5] and so too does the amount of time children spend in any form of care,[12] which may also influence the strength of the association between the childcare environment and obesity levels. Finally, it is possible that despite the influence of the childcare environment, parental or family-level factors, which are often targeted simultaneously, may exert a strong or stronger influence on children's weight status.[16]

In England, over 1 in 5 children are overweight or obese by the age of five.[17] We therefore sought to assess how the amount of time spent in childcare, and how the nutrition, physical activity and overall childcare environment are associated with anthropometric indicators (z-BMI score; waist-to-height ratio (WHR); sum of skinfold thickness (SST)) in a sample of UK 3-4-year-old children, adjusting for a range of family-level explanatory variables.

For peer review only - http://bmjopen.bmj.com/site/about/guidelines.xhtml

MATERIALS AND METHODS

Study design and recruitment

Data were from the "Studying Physical Activity in preschool-aged Children and their Environment (SPACE) Study", a cross-sectional childcare-based observational study[18]. Both preschool (state-run education) and nursery (privately-run care) childcare centres were purposively recruited in Cambridgeshire, UK, as funding, the built environment and care provided tend to differ by type.[18] Recruitment and data collection took place in January-July 2013; detailed information is available elsewhere.[18] Briefly, 88 centres in Cambridgeshire were approached to participate; 30 (34%) centre managers consented to participate. The University of Cambridge Psychology Ethics Committee provided ethical approval for the study (Pre.2012.68).

Data collection

Child Anthropometry

At each centre visit, one of three trained researchers recorded each child's sex; measured height to the nearest 0.1cm using a Leicester stadiometer; and weight to the nearest 0.1kg using Seca digital scales in light indoor clothes with shoes and socks removed. Abdominal waist circumference was measured to the nearest 0.5cm at the midpoint between the lower costal margin and the level of the anterior superior iliac crests. Up to three measures were taken (if there was a discrepancy of 1cm in the first two measures) using a Seca non-stretch tape measure next to the child's skin. Subscapular and tricep skinfolds were measured on the child's right side using a Holtain caliper (Holtain Ltd, United Kingdom) according to standard protocol. Compared to a 'gold standard' trainer, researcher mean differences in measurement was 0.1cm and 0.25-0.3mm for waist and skinfolds respectively. Equipment

For peer review only - http://bmjopen.bmj.com/site/about/guidelines.xhtml

was calibrated prior to commencing data collection, at the mid-way point and on completion of the study.

Assessment of Childcare Environment

A trained researcher assessed the physical activity and nutrition environment of each centre using the Environment and Policy Assessment and Observation (EPAO) instrument. Observation began when the first child arrived in the morning and continued until the end of the day, when the last child left,[19] as the EPAO protocol requires one full day to be spent observing all activities in the designated childcare classroom. Scoring is composed of two eight-item subscales for physical activity and nutrition. The physical activity subscales comprise: Active Opportunities, Sedentary Opportunities, Sedentary Environment, Portable Play Environment, Fixed Play Environment, Staff Physical Activity Behaviours, Physical Activity Training and Education, and Physical Activity Policy. Nutrition subscales comprise: Servings of fruits and vegetables; Whole grains; High-sugar/high-fat foods; Beverages; Staff nutrition behaviours; Nutrition environment; Nutrition training and education; and Nutrition policy.

As this tool was developed in the USA, a number of small amendments to the protocol and data collection template were made to ensure the tool was suitable in the UK context. This was done in consultation with the original development team to ensure the measure appropriately captured the physical activity and nutrition environments in UK childcare centres. These amendments did not change how the EPAO subscales for physical activity were scored; where preschools did not serve food, we used averages of available variables to derive the nutrition subscale scores.

For peer review only - http://bmjopen.bmj.com/site/about/guidelines.xhtml

Variable derivation

Outcome measures

Objectively measured height and weight were used to calculate children's BMI (weight[Kg]/ height^2[m]). This, combined with child's sex and age in months at measurement (calculated from parental reported date of child's birth), was used to derive a continuous BMI z-score, based on the British 1990 (UK90) growth reference charts.[20] Waist-to-height ratio (WHR) (waist [cm]/ height [cm]) was derived to assess central obesity, and the sum of subscapular and tricep skinfold thicknesses (SST) was derived as an indicator of subcutaneous fat[21].

Exposure variables

Parents were asked to provide information about their child's usual weekly childcare attendance using a specifically designed question:[18] "In a usual week, when does your child attend childcare? *Please only include care for your child taking part in SPACE and include regular formal and informal care (e.g. grandparents, friends etc.)*." Parents responded using free text, which we subsequently coded to derive the total number of reported hours children attended *formal* childcare during a usual week for analyses. Indicators of the overall childcare, nutrition and physical activity environments using EPAO (sub-scale) scores were also generated. According to standard EPAO scoring procedure, responses to questions across eight physical activity and up to eight nutrition sub-domains were summed to a possible maximum of 20 points per domain (where each question was worth 0-2 points). As not all UK childcare centres serve all meals (i.e. some only serve snacks, not lunch and 'tea' (at ~4pm)), two of the nutrition subscale scores (i.e. Whole grains; High-sugar/high-fat foods) were only relevant to nine centres here. Overall 'Physical Activity' and 'Nutrition' domain scores were therefore derived by averaging the subscale domain scores, i.e. 8 for physical activity and between 6 and 8 for nutrition (possible range 0-

For peer review only - http://bmjopen.bmj.com/site/about/guidelines.xhtml

20, higher score indicates more supportive environment). An overall 'EPAO score' by averaging the 8 physical activity and up to 8 nutrition subdomain scores (0-20) was also generated.

Additional confounding variables

A range of potential explanatory variables relating to the child's family environment were derived using data collected from a parental questionnaire, including child ethnicity (White British; White European; Other); maternal educational attainment (GCSEs, A Levels, NVQ/ Diploma; University degree; Higher degree); maternal self-report BMI; and hours per week mothers worked (Not employed; ≤20 hours; 21-35 hours; >35 hours). Where available, the latter three variables were also derived for fathers (with hours per week fathers worked categorised based on distribution as: <40 hours; 40-42 hours; >42 hours).

Statistical Analyses

All analyses were conducted using STATA 14/SE. We calculated descriptive statistics for included participants, and compared them to those excluded from analyses (i.e. those without valid anthropometric data) using independent t-tests and Pearson's chi^2.

For each of our three continuous outcome variables (z-BMI score, WHR, SST), a series of two-level mixed-effects linear regression analyses (level 1: child; level 2: childcare centre) were conducted to explore how weekly childcare attendance and practices relating to energy-balance behaviours in the childcare environment were related to children's anthropometric indices. Given children's age in months and sex are already taken into account when z-BMI score is derived, all WHR and SST analyses were adjusted for these variables. As children in the SPACE study were recruited or 'clustered' at the centre level, multi-level regression analyses were used to allow for both within- and between-setting variation in anthropometric

For peer review only - http://bmjopen.bmj.com/site/about/guidelines.xhtml

outcomes.[18,22] First, univariate analyses were conducted to assess how a) weekly childcare attendance (in hours), and b) the childcare environment relating to nutrition and physical activity, were associated with each of the three anthropometric indices. Multivariable analyses were then run, adjusting for family-level confounding variables.

Significant within-centre differences across the three outcome measures were identified. For example, 96% of the variation in z-BMI score was explained by within-centre (i.e. child-level) differences and only 4% of variation was explained by between-centre (i.e. childcare-level) differences [within-centre variation WHR: 73%; STT: 85%]. To further explore these differences, *post hoc* analyses were conducted to determine whether child-level socio-economic status (measured using maternal educational attainment) moderated the association between time spent in the childcare environment and each outcome. Based on previous evidence, we hypothesised that the relationship between a more favourable childcare environment and normal weight would be stronger (i.e. the gradient would be steeper) for children in lower income families. This was based on the assumption that favourable childcare environments would buffer the association between potentially negative home environments and overweight/ obesity in children from poorer socio-economic backgrounds, but that the childcare environment may be less important for children from higher income homes. Also, as fewer children provided SST data than z-BMI score and WHR, sensitivity analyses were conducted to limit our analyses to children providing complete case valid data for all three outcomes (n=151).

RESULTS

Participant Characteristics

For peer review only - http://bmjopen.bmj.com/site/about/guidelines.xhtml

Of the 245 children who assented to have anthropometric measurements taken, 196 children from 30 settings (n=15 preschools and n=15 nurseries) provided valid anthropometric, observational and questionnaire data (Table 1). Childcare centres have been described previously[23]; those centres who participated did not differ in terms of socio-economic status from those who declined to participate[18]. Children who provided complete case data (n=196) did not differ from those excluded by child's sex, age, weight status or ethnicity but were more likely to have higher educated mothers (higher degree: 38.3% vs. 22.2%, *p*=0.02).

For peer review only - http://bmjopen.bmj.com/site/about/guidelines.xhtml

Table 1 Descriptive characteristics of children included in analyses (SPACE study, 2013, N=196)

	Boys	Girls
**Child Characteristics**		
N (%)	100 (51)	96 (49)
Age (in months)	47.4 (5.2)	47.8 (4.9)
Ethnicity (N(%))		
White British	74 (74.0)	74 (77.1)
White other	9 (9.1)	9 (9.4)
Other/ mixed ethnicity	17 (17.0)	13 (13.5)
BMI z-score	0.52 (0.93)	0.36 (1.06)
Weight category[a] (N(%))		
Normal	82 (82.0)	79 (82.3)
Overweight/obese	18 (18.0)	17 (17.7)
Waist/ Height ratio	0.49 (0.1)	0.49 (0.1)
Centrally obese (N(%))	35 (46.7)	28 (40.6)
Sum of skinfolds[b] (cm)	14.5 (3.1)	16.0 (3.6)
Average weekly hours in childcare	23.3 (12.2)	21.8 (12.1)
**Maternal Characteristics**		
Age (in years)	37.6 (5.0)	37.4 (5.5)
BMI (in kg/m^2)	23.9 (4.2)	24.2 (4.7)
Education (N(%))		
GCSE/A-levels	25 (25.0)	34 (35.4)
Degree	29 (29.0)	33 (34.4)
Higher degree	46 (46.0)	29 (30.2)
Hours worked per week[c] (N(%))		
Not employed	26 (27.4)	24 (25.8)
<20 hours	19 (20.0)	14 (15.1)
21-35 hours	28 (29.5)	31 (33.3)
>35 hours	22 (23.2)	24 (25.8)
**Paternal Characteristics[e]**		
Age (in years)	39.2 (5.6)	39.4 (8.0)
BMI (in kg/m^2)	25.2 (3.5)	25.9 (3.2)
Paternal education (N(%))		
GCSE/A-levels	23 (23)	20 (24.1)
Degree	24 (24)	27 (32.5)
Higher degree	43 (43)	36 (43.4)
Hours worked per week[c] (N(%))		
<40 hours	26 (29.8)	30 (35.3)
40-42 hours	30 (35.6)	30 (36.6)
>42 hours	30 (34.5)	22 (26.8)

All values mean(SD) unless stated otherwise; BMI: Body mass index; a: Weight category derived using the International Task Force on Obesity cut points; GCSE: General Certificate of Secondary Education; A-levels: Advanced Levels; b: n=80 boys and n=71 girls; c: categorized based on distribution; c: maternal employment: n=95 for boys, n=93 for girls; d: paternal variables available for n=169-173 children depending on variable

For peer review only - http://bmjopen.bmj.com/site/about/guidelines.xhtml

Childcare Environment

Domain subscale and total scores for the EPAO are shown in Table 2. Across childcare centres, the mean total EPAO score (including eight physical activity and up to eight nutrition domains) was 11.2 (SD: 1.0, range: 8.5 - 13.5). The average Physical Activity subscale score was 10.8 (1.5, 7.4-13.8) and average Nutrition subscale score was 11.7 (1.6, 8.8-14.3).

Table 2 EPAO average domain subscale and total scores in the SPACE study

Nutrition sub-domains[a]	Mean (SD)	Range	Physical Activity sub-domains[b]	Mean (SD)	Range
Servings: fruits and vegetables	13.4 (4.1)	6.7-20.0	Active Opportunities	15.3 (3.8)	6.7 – 20.0
Servings: Whole grains[c]	3.3 (3.3)	0-10.0	Sedentary Opportunities	11.7 (4.5)	6.7 – 20.0
Servings: High-sugar/high-fat foods[d]	14.5 (0.9)	14.0 –16.0	Sedentary Environment	12.7 (4.1)	6.7 – 20.0
Servings: Beverages	9.7 (2.4)	5.0 - 16.3	Portable Play Environment	10.7 (3.1)	5.7 - 17.1
Staff nutrition behaviours	10.1 (2.8)	3.3 -15.0	Fixed Play Environment	11.8 (2.8)	7.5 - 16.3
Nutrition environment	15.4 (3.0)	6.7 – 20.0	Staff PA Behaviours	14.3 (2.5)	8.0 – 20.0
Nutrition training and education	10.6 (3.1)	4.0 – 18.0	PA Training and Education	4.8 (4.0)	0 - 15.0
Nutrition policy	12.6 (6.2)	6.7 – 20.0	PA Policy	7.0 (6.8)	0 - 20.0
**Total Domain Scores[b]**					
Average Nutrition domain score[e]	11.7 (1.6)	8.8 - 14.3	Average Physical Activity domain score[f]	10.8 (1.5)	7.4 - 13.8
**Total EPAO Scores[b]**					
Average total EPAO score[g]	11.2 (1.0)	8.5 - 13.5			

EPAO: Environment and Policy Assessment and Observation tool; a: n=30 unless stated; b: n=30; c: centres n=9; d: centres n=8; e: average of up to 8 subdomain scores f: includes 8 subdomain scores; g: average of up to 8 nutrition and 8 physical activity domain scores

For peer review only - http://bmjopen.bmj.com/site/about/guidelines.xhtml

Association between childcare environment and children's anthropometric indices

In unadjusted analyses, there was no significant association between the number of hours a child spent in care or energy-balance childcare practices and preschoolers' anthropometric indices (Table 3). These findings remained unchanged after adjusting for family-level variables; as expected, several family-level variables were independently associated with the outcomes of interest in adjusted models (see supplementary Table S1).

We found no significant interactions in *post hoc* analyses, designed to determine whether child-level socio-economic status (measured using maternal educational attainment) moderated the association between time spent in the childcare environment and our three outcome measures (data not shown). In sensitivity analyses, including only children who provided all three outcome measures (n=151) did not significantly influence our findings (data not shown).

For peer review only - http://bmjopen.bmj.com/site/about/guidelines.xhtml

Table 3 Associations between childcare environment and preschool-aged children's anthropometric indices in the SPACE study

	Exposure measures β [95% CI]							
	Weekly hours in care		EPAO PA score		EPAO Nutrition score		EPAO total score	
**Continuous Outcomes**	Unadjusted	Adjusted[a]	Unadjusted	Adjusted[a]	Unadjusted	Adjusted[a]	Unadjusted	Adjusted[a]
BMI z-score[b]	-0.00	-0.01	-0.02	-0.01	0.01	-0.01	-0.00	-0.00
	[-0.02,0.01]	[-0.02,0.00]	[-0.12,0.09]	[-0.12,0.11]	[-0.09,0.10]	[-0.11,0.09]	[-0.01,0.01]	[-0.01,0.01]
Waist to height ratio[b,c]	-0.00	-0.00	0.01	0.00	-0.00	-0.00	0.00	0.00
	[-0.00,0.00]	[-0.00,0.00]	[-0.01,0.02]	[-0.01,0.02]	[-0.01,0.01]	[-0.01,0.01]	[-0.00,0.00]	[-0.00,0.00]
Sum of skinfold[c,d]	0.02	0.03	0.03	0.18	-0.03	-0.18	0.00	0.00
	[-0.02,0.07]	[-0.01,0.08]	[-0.44,0.50]	[-0.30,0.67]	[-0.45,0.40]	[-0.61,0.26]	[-0.05,0.05]	[-0.05,0.05]

EPAO: Environment and Policy Assessment and Observation tool; a: model adjusted for child ethnicity, maternal BMI, maternal educational attainment and maternal working hours; b: n=196; c: model also includes child sex and age in months; d: n=144

For peer review only

CONCLUSIONS

We found that childcare attendance and energy-balance practices in the childcare environment do not appear to be associated with UK preschool-aged children's anthropometric indices. Given large within-centre (or child-level) variation in our outcome measures, it is perhaps unsurprising that we did not identify associations between time spent in the childcare environment and children's anthropometry here. As shown previously, family-level factors were independently associated with children's z-BMI score. Although children spend increasing amounts of time in out-of-home care and the childcare environment is frequently the focus of intervention efforts to prevent or reduce early childhood obesity,[24] family-level factors also remain important and warrant equal attention when considering obesity prevention strategies for young children.

How this work compares to previous studies

Cross-sectionally, we did not identify any associations between time spent in care and children's anthropometry. This is in accordance with previous studies conducted in low-income Latino 4-year-olds[25] and 2-5-year olds in Australia[26] which also failed to find an association between time spent in care and adiposity. Interestingly, in the latter Australian study, which reported a similar prevalence of overweight and obesity to our sample (16.7 vs 17.9 %), they identified maternal education as the sole significant predictor of child's weight status.[26] Although family level factors did not confound the association here (i.e. no association was found before or after adjustment), several were independently associated with BMI z-score, significantly attenuating the relationship between exposure and outcome. This suggests that as found previously,[16] family level factors may influential to children's weight status, regardless of childcare attendance.

Within the SPACE study, there was relatively wide heterogeneity in childcare environments within small geographical areas. EPAO scores in this study were similar to those seen previously in US and Dutch studies,[23] which suggests there were no obvious differences between these UK and other childcare environments. However, across and between countries differences may exist in how 'healthy' childcare

For peer review only - http://bmjopen.bmj.com/site/about/guidelines.xhtml

environments are, and there may also be variation across physical activity and nutrition environments within the same childcare environment (i.e. good nutrition, poor physical activity and vice versa). Taken together, the definition of a 'healthy' environment is likely to be relatively heterogeneous, potentially preventing identification of consistent associations.

It could be hypothesised that childcare in earlier childhood (i.e. infancy), rather than during the preschool-period (as assessed here), is more important for the development of children's adiposity. Indeed, work conducted in prospective cohorts suggests that early childcare attendance may be associated with children's weight status: Black and colleagues noted that early informal care (before 3 years) was associated with increased risk of overweight/obesity, and that higher childcare attendance, especially starting before 1 year, also increased overweight/obesity risk.[11] Cumulative exposure to centre-based care up to the age of 4 was also associated with higher odds of overweight/obesity in later childhood, i.e. at 4-10 years of age,[8] in Canadian children. As such, these longitudinal associations appear to be dependent on the timing and level of exposure to childcare.[11,27] Greater childcare attendance in very early childhood appears to be associated with childhood obesity, but later childcare attendance does not show such associations.[11] As no data on previous childcare attendance patterns were collected in this study, we were unable to test this hypothesis here.

It is conceivable that bias or residual confounding, in addition to our sample size, limited our ability to find associations; it is also possible that a lack of power (i.e. low β) resulted in a type 2 error, or us failing to find an association where one existed. This said, we were able to detect relatively small associations between the outcome and family level factors (e.g. maternal BMI: β co-efficient=0.05), and had suitable heterogeneity in our exposure measures to do so. Previous studies have identified associations with a range of time-based exposures assessed over longer periods of time (i.e. 10 hours/week increments;[28] each additional 30 days exposure to care[9] during the first year of life) or simply by the type of care children were exposed to (i.e. childcare vs. informal vs. parental care[8,12]).[27] This variation in exposure and outcome measures is

For peer review only - http://bmjopen.bmj.com/site/about/guidelines.xhtml

therefore also likely to account for differing findings between childcare-related factors and children's anthropometric indices.

This is to our knowledge one of the few studies to go beyond exploration of the association between time spent in care and anthropometry, to assess how elements within the childcare environment (i.e. nutrition and physical activity practices) are related to a children's anthropometric indices. In a previous study using this same sample of children, we found a similar lack of association between preschoolers' physical activity and the childcare environment.[23] It is possible that this is due in part to a fairly standardised system of care in the early years in the UK: all childcare centres are mandated to follow the statutory Early Years Foundation Stage (EYFS) framework,[29] a curriculum against which children's development is judged at age 2 and age 5 (after their first year in primary school). A key tenant of this is that children must "be helped to understand the importance of physical activity, and to make healthy choices in relation to food." (Pg 5;[29]) With this core mandate to promote healthy EBRBs, and with high levels of children attending childcare, childcare settings in the UK appear well placed to ensure all children receive the best start in life, regardless of socio-demographic factors.

To date, much of the research in this area has focused on the out-of-home care environment, and strategies to encourage positive health behaviours and weight in preschool-aged children are increasingly centred on the childcare environment, particularly in the UK.[24] However, very little intervention research has been conducted in the UK childcare setting, with this and previous research[23] suggesting that the UK childcare environment does not appear to be associated with preschool-aged children's anthropometric and physical activity outcomes. Nevertheless, areas for improvement within the childcare environment likely exist, and in the absence of intervention evidence, a more targeted approach, focusing on specific areas within the childcare environment (e.g. improving dietary provision[15]), may still benefit children's health behaviours. Moreover, children continue to receive a significant proportion of their care in the home environment, with family-level factors tending to be associated with young children's anthropometric outcomes, independent

For peer review only - http://bmjopen.bmj.com/site/about/guidelines.xhtml

of childcare attendance.[16] This is confirmed by a recent review that found that informal care, in contrast to formal centre-based care, was associated with increased weight in children 6 years and under in a number of prospective studies.[27] Family-level and home-based factors therefore continue to warrant substantial attention when considering obesity prevention strategies for young children.

Strengths and limitations

We used objective measures of UK preschoolers' anthropometric indices to provide novel information about how attendance and the childcare environment are associated with children's anthropometric indices. Although this was a relatively small UK childcare-based sample, both our outcomes and exposures were normally distributed, and provided us with sufficient heterogeneity to explore our research questions. Although derivation of one of the exposure measures (i.e. the Nutrition domain score) differed from previous studies, limiting the number of sub-scale scores used (to 6 rather than taking an average of up to 8) did not influence our findings. By using hierarchical regression analysis, we were also able to take account of study design and potential clustering at the centre-level, thus increasing our power to detect (small) significant differences in our outcomes. We also adjusted for a number of family-level factors known to be related to children's anthropometry but we did not have a measure of each child's birth-weight to account for children's individual growth trajectories. As with several studies in this area,[27] this work was based on childcare-based sample. We were therefore unable to assess the influence of no or only informal care, in addition to centre-based care, on children's weight-related outcomes.

For peer review only

Children were drawn from settings recruited from the top tertile of Index of Multiple Deprivation (IMD) scores in England.[18] As there were no differences in IMD scores of the centres that did and did not participate in the SPACE study,[23] children included here are likely representative of the wider eligible population. Children's individual socio-economic circumstances (i.e. maternal educational attainment) varied within this sample, which is important as children from both lower and higher socio-economic backgrounds may be at risk of higher BMI.[12,13,17] It is not therefore not clear what how generalizable

For peer review only - http://bmjopen.bmj.com/site/about/guidelines.xhtml

our findings are. However, we did not see evidence of a moderating effect of children's socio-economic circumstances on the exposure-outcome relationship, suggesting that the association may be consistent across socio-economic strata. Just under one-fifth (18%) of children were classified as overweight or obese, which is slightly below the national UK average for 5-year old children (22.5% in 2013/14).[17] Children were predominantly White (British/ European) (85%), which is in line with the UK average (86% in 2011).[30] Work is however required to determine whether similar findings are apparent in differing minority populations across the UK.

For peer review only

CONCLUSIONS

We found no significant association between childcare attendance, or the nutrition and physical activity childcare environment, and children's anthropometric outcomes. We did however identify large within-centre (or between-child) variation in outcomes, and as found previously, family-level factors were associated with z-BMI scores. Although childcare-based interventions are increasingly the focus for promoting healthy weight and EBRBs in pre-schoolers, they tend to show small effects that are not sustained over time. Targeting family-level factors, as an adjunct or alternative to centre-based interventions, should not therefore be disregarded, and may also yield success in promoting health weight in preschool-aged children.

For peer review only

ACKNOWLEDGMENTS

We thank all children and their parents who participated in the SPACE study. In addition, we thank members of the MRC Epidemiology Unit for their assistance: to members of the field team who conducted data collection, to Kate Westgate and Stefanie Hollidge from the physical activity technical team for the accelerometer data processing, and to Dr Emanuella De Lucia Rolfe and Richard Powell for processing the anthropometric data.

CONTRIBUTORSHIP

KH and EvS conceived of the SPACE study. KH collected the data, conducted the analyses and drafted the manuscript. KH, SBN and EvS interpreted the results and critically revised the manuscript. All authors approved the final manuscript and agree to be accountable for all aspects of the work.

FUNDING

This work was conducted by the Medical Research Council [Unit Programme number MC_UU_12015/7] and the Centre for Diet and Activity Research (CEDAR), a UKCRC Public Health Research Centre of Excellence. Funding from the British Heart Foundation, Cancer Research UK, Economic and Social Research Council, Medical Research Council, the National Institute for Health Research, and the Wellcome Trust, under the auspices of the UK Clinical Research Collaboration, is gratefully acknowledged (CEDAR grant numbers: ES/G007462/1; 087636/Z/08/Z; MR/K023187/1). KH is funded by the Wellcome Trust (107337/Z/15/Z). SBN is funded by NIH and the Robert Wood Johnson Foundation.

COMPETING INTERESTS

All authors have completed the ICMJE uniform disclosure form at http://www.icmje.org/coi_disclosure.pdf (available on request from the corresponding author) and declare that (1) they have support from a number of grants outlined above; (2) all authors have no relationships with any organisations that might have an interest in the submitted work in the previous 3 years; (3) their spouses, partners or children have no financial relationships that may be relevant to the submitted work and (4) have no non-financial interests that may be relevant to the submitted work.

ETHICS APPROVAL

Ethical approval was granted by The University of Cambridge Psychology Ethics Committee for the study (Pre.2012.68).

For peer review only - http://bmjopen.bmj.com/site/about/guidelines.xhtml

DATA SHARING STATEMENT

Unpublished data regarding children's health behaviours, socio-economic circumstances and parental behaviours are available for the SPACE study. The anonymised data set and statistical code used for analyses are available from the corresponding author on request. Model parameters are outlined in the 'Methods' section. Participants gave assent and parents provided informed consent for study participation, but the presented data are anonymised and risk of identification is very low.

For peer review only

REFERENCES

1 de Onis M, Blossner M, Borghi E. Global prevalence and trends of overweight and obesity among preschool children. *Am J Clin Nutr* 2010;**92**:1257–64. doi:10.3945/ajcn.2010.29786.1

2 Rokholm B, Baker J, Sørensen T. The levelling off of the obesity epidemic since the year 1999 - a review of evidence and perspectives. *Obes Rev* 2010;**11**:835–46. doi:10.1111/j.1467-789X.2010.00810.x

3 Lakshman R, Elks CE, Ong KK. Childhood Obesity. *Circulation* 2012;**126**:1770–9. doi:10.1161/CIRCULATIONAHA.111.047738

4 World Health Organisation. Obesity and overweight. WHO Fact sheet N°311. 2013.http://www.who.int/mediacentre/factsheets/fs311/en/

5 OECD. Equity and Quality in Education. 2012. doi:10.1787/9789264130852-en

6 Trost SG, Ward DS, Senso M. Effects of child care policy and environment on physical activity. *Med Sci Sport Exerc* 2010;**42**:520–5. doi:10.1249/MSS.0b013e3181cea3ef

7 Gubbels JS, Gerards SMPL, Kremers SPJ. Use of food practices by childcare staff and the association with dietary intake of children at childcare. *Nutrients* 2015;**7**:2161–75. doi:10.3390/nu7042161

8 Geoffroy M-C, Power C, Touchette E, *et al.* Childcare and overweight or obesity over 10 years of follow-up. *J Pediatr* 2013;**162**:753–758.e1. doi:10.1016/j.jpeds.2012.09.026

9 Benjamin Neelon SE, Schou Andersen C, Schmidt Morgen C, *et al.* Early child care and obesity at 12 months of age in the Danish National Birth Cohort. *Int J Obes* 2015;**39**:33–8. doi:10.1038/ijo.2014.173

10 Sisson SB, Li J, Stoner JA, *et al.* Obesogenic environments in tribally-affiliated childcare centers and corresponding obesity rates in preschool children. *Prev Med Reports* 2016;**3**:151–8. doi:10.1016/j.pmedr.2016.01.003

11 Black L, Matvienko-Sikar K, Kearney PM. The association between childcare arrangements and risk of overweight and obesity in childhood: a systematic review. *Obes Rev* 2017;**18**:1170–90. doi:10.1111/obr.12575

For peer review only - http://bmjopen.bmj.com/site/about/guidelines.xhtml

12 Pearce A, Li L, Abbas J, *et al.* Is childcare associated with the risk of overweight and obesity in the early years? Findings from the UK Millennium Cohort Study. *Int J Obes* 2010;**34**:1160–8. doi:10.1038/ijo.2010.15

13 Nazarov Z, Rendall M. Differences by mother's education in the effect of childcare on child obesity. *Econ Lett* 2014;**124**:286–9. doi:10.1016/j.pestbp.2011.02.012.Investigations

14 Zhou YE, Emerson JS, Levine RS, *et al.* Childhood obesity prevention interventions in childcare settings: Systematic review of randomized and nonrandomized controlled trials. *Am J Heal Promot* 2014;**28**:92–104. doi:10.4278/ajhp.121129-LIT-579

15 Neelon SEB, Burgoine T, Hesketh KR, *et al.* Nutrition practices of nurseries in England: Comparison with national guidelines. *Appetite* Published Online First: November 2014. doi:10.1016/j.appet.2014.11.002

16 Hawkins SS, Cole TJ, Law C. An ecological systems approach to examining risk factors for early childhood overweight: findings from the UK Millennium Cohort Study. *J Epidemiol Community Health* 2009;**63**:147–55. doi:10.1136/jech.2008.077917

17 NHS Digital. National Child Measurement Programme - England, 2014-15. Natl. Child Meas. Program. 2015.

18 Hesketh KR, Griffin SJ, van Sluijs EMF. UK Preschool-aged children's physical activity levels in childcare and at home: a cross-sectional exploration. *Int J Behav Nutr Phys Act* 2015;**12**:123. doi:10.1186/s12966-015-0286-1

19 Ammerman AS, Ward DS, Benjamin SE, *et al.* An Intervention to Promote Healthy weight: Nutrition and Physical Activity Self-Assessment for Child Care (NAP SACC) theory and design. *Prev Chronic Dis* 2007;**4**:A67.http://www.ncbi.nlm.nih.gov/pubmed/17572971

20 Cole TJ, Freeman J V, Preece MA. Body mass index reference curves for the UK, 1990. *Arch Dis Child* 1995;**73**:25–9. doi:10.1136/adc.73.1.25

21 Freedman DS, Katzmarzyk PT, Dietz WH, *et al.* Relation of body mass index and skinfold thicknesses to cardiovascular disease risk factors in children : the Bogalusa Heart Study 1 – 4. *Am J*

For peer review only - http://bmjopen.bmj.com/site/about/guidelines.xhtml

Clin Nutr 2009;**90**:210–6. doi:10.3945/ajcn.2009.27525.1

22 Goldstein H. *Multilevel Statistical Models*. 4th ed. Wiley-Blackwell 2010.

23 Hesketh KR, van Sluijs EMF. Features of the UK childcare environment and associations with preschooler's in-care physical activity. *Prev Med Reports* 2016;**3**:53–7. doi:10.1016/j.pmedr.2015.12.004

24 Department of Health. Childhood Obesity: A Plan for Action. London, UK: 2016. https://www.gov.uk/government/uploads/system/uploads/attachment_data/file/546588/Childhood_obesity_2016__2__acc.pdf

25 Zahir N, Heyman MB, Wojcicki JM. No association between childcare and obesity at age 4 in low-income Latino children. *Pediatr Obes* 2012;**8**:E24-28. doi:10.1111/j.20I47-6T310.2012.Y00125.x

26 Wolfenden L, Hardy LL, Wiggers J, *et al.* Prevalence and socio-demographic associations of overweight and obesity among children attending child-care services in rural and regional Australia. *Nutr Diet* 2011;**68**:15–20. doi:10.1111/j.1747-0080.2010.01487.x

27 Alberdi G, McNamara AE, Lindsay KL, *et al.* The association between childcare and risk of childhood overweight and obesity in children aged 5 years and under: a systematic review. *Eur J Pediatr* 2016;**175**:1277–94. doi:10.1007/s00431-016-2768-9

28 Benjamin SE, Rifas-Shiman SL, Taveras EM, *et al.* Early child care and adiposity at ages 1 and 3 years. *Pediatrics* 2009;**124**:555–62. doi:10.1542/peds.2008-2857

29 Department for Education. Early Years Foundation Stage (EYFS). United Kingdom: 2012. hhttp://www.education.gov.uk/aboutdfe/statutory/g00213120/eyfs-statutory-framework

30 Office of National Statistics. Ethnicity and National Identity in England and Wales: 2011. London, UK: 2012. http://www.ons.gov.uk/peoplepopulationandcommunity/culturalidentity/ethnicity/articles/ethnicityandnationalidentityinenglandandwales/2012-12-11

For peer review only - http://bmjopen.bmj.com/site/about/guidelines.xhtml

Supplementary Table S1i: Associations between childcare attendance and environment and BMI z-score (n=196)

	BMI z-score - β [95% CI]							
	Unadjusted	Adjusted	Unadjusted	Adjusted	Unadjusted	Adjusted	Unadjusted	Adjusted
Constant	0.51 [0.22,0.81]	-0.35 [-1.27,0.56]	0.62 [-0.52,1.77]	-0.50 [-2.02,1.02]	0.36 [-0.82,1.53]	-0.45 [-1.91,1.00]	0.58 [-1.11,2.26]	-0.39 [-2.33,1.56]
**EXPOSURE**								
Weekly hours in care	-0.00 [-0.02,0.01]	-0.01 [-0.02,0.00]						
PA score			-0.02 [-0.12,0.09]	-0.01 [-0.12,0.11]				
Nutrition score					0.01 [-0.09,0.10]	-0.01 [-0.11,0.09]		
EPAO total score							-0.00 [-0.01,0.01]	-0.00 [-0.01,0.01]
**Child factors**								
Ethnicity (ref: white)								
White European		-0.06 [-0.56,0.44]		-0.10 [-0.61,0.40]		-0.10 [-0.61,0.40]		-0.10 [-0.61,0.41]
Other/ mixed		0.09 [-0.30,0.49]		0.05 [-0.35,0.45]		0.05 [-0.35,0.44]		0.05 [-0.34,0.45]
**Maternal Factors**								
Maternal BMI		0.05** [0.02,0.08]		0.05*** [0.02,0.08]		0.05*** [0.02,0.08]		0.05*** [0.02,0.08]
Maternal education (ref: GCSE/ A-levels)								
Degree		-0.49** [-0.83,-0.15]		-0.52** [-0.86,-0.18]		-0.52** [-0.86,-0.18]		-0.52** [-0.86,-0.18]
Higher degree		0.04 [-0.31,0.39]		0.01 [-0.34,0.35]		0.01 [-0.33,0.36]		0.01 [-0.33,0.35]
Maternal working hours (ref: not employed)								
<20 hours		-0.37 [-0.79,0.04]		-0.40 [-0.81,0.02]		-0.40 [-0.81,0.02]		-0.40 [-0.81,0.02]
21-35 hours		-0.01 [-0.38,0.36]		-0.06 [-0.42,0.30]		-0.06 [-0.41,0.30]		-0.05 [-0.41,0.31]
>35 hours		0.20 [-0.21,0.61]		0.09 [-0.29,0.47]		0.09 [-0.29,0.47]		0.09 [-0.29,0.47]

95% confidence intervals in brackets; * $p < 0.05$, ** $p < 0.01$, *** $p < 0.001$

For peer review only

Supplementary Table S1ii: Associations between childcare attendance and environment and Waist to Height Ratio (n=188)

	Waist to Height Ratio (WHR) - β [95% CI]							
	Unadjusted	Adjusted	Unadjusted	Adjusted	Unadjusted	Adjusted	Unadjusted	Adjusted
Constant	0.48***<br>[0.36,0.60]	0.50***<br>[0.36,0.65]	0.42***<br>[0.25,0.59]	0.44***<br>[0.24,0.64]	0.51***<br>[0.34,0.67]	0.53***<br>[0.35,0.71]	0.44***<br>[0.23,0.65]	0.47***<br>[0.24,0.71]
**EXPOSURE**								
Weekly hours in care	-0.00<br>[-0.00,0.00]	-0.00<br>[-0.00,0.00]						
PA score			0.01<br>[-0.01,0.02]	0.00<br>[-0.01,0.02]				
Nutrition score					-0.00<br>[-0.01,0.01]	-0.00<br>[-0.01,0.01]		
Total EPAO score							0.00<br>[-0.00,0.00]	0.00<br>[-0.00,0.00]
**Child factors**								
Sex	0.01<br>[-0.02,0.03]	0.01<br>[-0.02,0.03]	0.01<br>[-0.02,0.03]	0.01<br>[-0.02,0.03]	0.01<br>[-0.02,0.03]	0.01<br>[-0.02,0.03]	0.01<br>[-0.02,0.03]	0.01<br>[-0.02,0.03]
Age in months	0.00<br>[-0.00,0.00]	0.00<br>[-0.00,0.00]	0.00<br>[-0.00,0.00]	0.00<br>[-0.00,0.00]	0.00<br>[-0.00,0.00]	0.00<br>[-0.00,0.00]	0.00<br>[-0.00,0.00]	0.00<br>[-0.00,0.00]
Ethnicity (ref: white)								
White European		0.01<br>[-0.04,0.06]		0.00<br>[-0.05,0.05]		0.01<br>[-0.04,0.05]		0.00<br>[-0.04,0.05]
Other/ mixed		0.00<br>[-0.04,0.04]		-0.00<br>[-0.04,0.04]		-0.00<br>[-0.04,0.04]		-0.00<br>[-0.04,0.04]
**Maternal Factors**								
Maternal BMI		-0.00<br>[-0.00,0.00]		-0.00<br>[-0.00,0.00]		-0.00<br>[-0.00,0.00]		-0.00<br>[-0.00,0.00]
Maternal education (ref: GCSE/ A-levels)								
Degree		0.00<br>[-0.03,0.03]		0.00<br>[-0.03,0.03]		-0.00<br>[-0.03,0.03]		0.00<br>[-0.03,0.03]
Higher degree		-0.00<br>[-0.03,0.03]		-0.00<br>[-0.03,0.03]		-0.00<br>[-0.04,0.03]		-0.00<br>[-0.04,0.03]
Maternal working hours (ref: not employed)								
<20 hours		0.01<br>[-0.03,0.05]		0.00<br>[-0.04,0.04]		0.00<br>[-0.04,0.04]		0.00<br>[-0.04,0.04]
21-35 hours		0.01<br>[-0.02,0.05]		0.01<br>[-0.03,0.04]		0.01<br>[-0.03,0.04]		0.01<br>[-0.03,0.04]
> 35 hours		-0.00<br>[-0.04,0.03]		-0.01<br>[-0.05,0.02]		-0.01<br>[-0.05,0.02]		-0.01<br>[-0.05,0.02]

95% confidence intervals in brackets; * $p < 0.05$, ** $p < 0.01$, *** $p < 0.001$

For peer review only

For peer review only - http://bmjopen.bmj.com/site/about/guidelines.xhtml

Supplementary Table S1iii: Associations between childcare attendance and environment and sum of skinfolds thickness (n=144)

	Sum of Skinfolds - β [95% CI]							
	Unadjusted	Adjusted	Unadjusted	Adjusted	Unadjusted	Adjusted	Unadjusted	Adjusted
Constant	15.12***	11.66***	15.71***	10.17*	16.38***	14.30***	15.96***	12.09*
	[9.80,20.44]	[6.03,17.30]	[8.20,23.23]	[2.07,18.27]	[9.12,23.64]	[6.81,21.78]	[6.75,25.17]	[2.43,21.76]
**EXPOSURE**								
Weekly hours in care	0.02	0.03						
	[-0.02,0.07]	[-0.01,0.08]						
PA score			0.03	0.18				
			[-0.44,0.50]	[-0.30,0.67]				
Nutrition score					-0.03	-0.18		
					[-0.45,0.40]	[-0.61,0.26]		
EPAO total score							0.00	0.00
							[-0.05,0.05]	[-0.05,0.05]
**Child factors**								
Sex	1.18*	1.41**	1.25*	1.53**	1.24*	1.52**	1.25*	1.53**
	[0.18,2.19]	[0.47,2.35]	[0.23,2.27]	[0.57,2.49]	[0.22,2.26]	[0.57,2.48]	[0.23,2.27]	[0.57,2.49]
Age in months	-0.02	-0.05	-0.03	-0.06	-0.03	-0.06	-0.03	-0.06
	[-0.13,0.09]	[-0.16,0.05]	[-0.14,0.08]	[-0.17,0.05]	[-0.14,0.08]	[-0.17,0.05]	[-0.14,0.08]	[-0.17,0.05]
Ethnicity (ref: white)								
White European		-0.18		-0.35		-0.20		-0.25
		[-1.94,1.58]		[-2.17,1.48]		[-2.00,1.61]		[-2.07,1.58]
Other/ mixed		-0.04		-0.01		0.08		0.10
		[-1.51,1.42]		[-1.51,1.50]		[-1.40,1.55]		[-1.39,1.59]
**Maternal Factors**								
Maternal BMI		0.27***		0.27***		0.27***		0.27***
		[0.15,0.40]		[0.15,0.40]		[0.15,0.40]		[0.14,0.40]
Maternal education (ref: GCSE/ A-levels)								
Degree		-1.61**		-1.46*		-1.49*		-1.49*
		[-2.81,-0.41]		[-2.67,-0.24]		[-2.70,-0.28]		[-2.71,-0.28]
Higher degree		-0.27		0.13		0.16		0.09
		[-1.54,1.00]		[-1.13,1.40]		[-1.11,1.44]		[-1.18,1.35]
Maternal working hours (ref: not employed)								
<20 hours		-1.03		-0.93		-0.93		-0.90
		[-2.44,0.37]		[-2.36,0.50]		[-2.36,0.50]		[-2.33,0.53]
21-35 hours		-0.30		-0.01		0.07		0.04
		[-1.64,1.04]		[-1.34,1.31]		[-1.24,1.39]		[-1.29,1.37]
>35 hours		-0.36		-0.05		-0.04		-0.05
		[-1.82,1.09]		[-1.42,1.32]		[-1.41,1.33]		[-1.42,1.33]

95% confidence intervals in brackets; * $p < 0.05$, ** $p < 0.01$, *** $p < 0.001$

For peer review only

For peer review only - http://bmjopen.bmj.com/site/about/guidelines.xhtml

STROBE 2007 (v4) Statement—Checklist of items that should be included in reports of *cross-sectional studies*

Section/Topic	Item #	Recommendation	Reported on page #
**Title and abstract**	1	(*a*) Indicate the study's design with a commonly used term in the title or the abstract	2
		(*b*) Provide in the abstract an informative and balanced summary of what was done and what was found	2
**Introduction**			
Background/rationale	2	Explain the scientific background and rationale for the investigation being reported	3-5
Objectives	3	State specific objectives, including any prespecified hypotheses	5
**Methods**			
Study design	4	Present key elements of study design early in the paper	6
Setting	5	Describe the setting, locations, and relevant dates, including periods of recruitment, exposure, follow-up, and data collection	6-7
Participants	6	(*a*) Give the eligibility criteria, and the sources and methods of selection of participants	6
Variables	7	Clearly define all outcomes, exposures, predictors, potential confounders, and effect modifiers. Give diagnostic criteria, if applicable	6-8
Data sources/ measurement	8*	For each variable of interest, give sources of data and details of methods of assessment (measurement). Describe comparability of assessment methods if there is more than one group	6-8
Bias	9	Describe any efforts to address potential sources of bias	
Study size	10	Explain how the study size was arrived at	6/ 10
Quantitative variables	11	Explain how quantitative variables were handled in the analyses. If applicable, describe which groupings were chosen and why	6--9
Statistical methods	12	(*a*) Describe all statistical methods, including those used to control for confounding	9
		(*b*) Describe any methods used to examine subgroups and interactions	9-10
		(*c*) Explain how missing data were addressed	10
		(*d*) If applicable, describe analytical methods taking account of sampling strategy	9/10
		(*e*) Describe any sensitivity analyses	9/10
**Results**			

For peer review only

Participants	13*	(a) Report numbers of individuals at each stage of study—eg numbers potentially eligible, examined for eligibility, confirmed eligible, included in the study, completing follow-up, and analysed	10
		(b) Give reasons for non-participation at each stage	10
		(c) Consider use of a flow diagram	-
Descriptive data	14*	(a) Give characteristics of study participants (eg demographic, clinical, social) and information on exposures and potential confounders	12
		10	10
Outcome data	15*	Report numbers of outcome events or summary measures	10/11
Main results	16	(*a*) Give unadjusted estimates and, if applicable, confounder-adjusted estimates and their precision (eg, 95% confidence interval). Make clear which confounders were adjusted for and why they were included	14/15
		(*b*) Report category boundaries when continuous variables were categorized	-
		(*c*) If relevant, consider translating estimates of relative risk into absolute risk for a meaningful time period	-
Other analyses	17	Report other analyses done—eg analyses of subgroups and interactions, and sensitivity analyses	14
**Discussion**			
Key results	18	Summarise key results with reference to study objectives	16
Limitations	19	Discuss limitations of the study, taking into account sources of potential bias or imprecision. Discuss both direction and magnitude of any potential bias	20/21
Interpretation	20	Give a cautious overall interpretation of results considering objectives, limitations, multiplicity of analyses, results from similar studies, and other relevant evidence	22
Generalisability	21	Discuss the generalisability (external validity) of the study results	21
**Other information**			
Funding	22	Give the source of funding and the role of the funders for the present study and, if applicable, for the original study on which the present article is based	23

*Give information separately for cases and controls in case-control studies and, if applicable, for exposed and unexposed groups in cohort and cross-sectional studies.

Note: An Explanation and Elaboration article discusses each checklist item and gives methodological background and published examples of transparent reporting. The STROBE checklist is best used in conjunction with this article (freely available on the Web sites of PLoS Medicine at http://www.plosmedicine.org/, Annals of Internal Medicine at http://www.annals.org/, and Epidemiology at http://www.epidem.com/). Information on the STROBE Initiative is available at www.strobe-statement.org.

For peer review only

BMJ Open

How does the UK childcare energy-balance environment influence 3-to-4-year-olds' anthropometry? A cross-sectional exploration

Journal:	*BMJ Open*
Manuscript ID	bmjopen-2018-021520.R1
Article Type:	Research
Date Submitted by the Author:	27-Mar-2018
Complete List of Authors:	Hesketh, Kathryn R.; UCL Institute of Child Health, Population, Policy and Practice; University of Cambridge, CEDAR and MRC Epidemiology Unit<br>Benjamin Neelon, Sara E.; Johns Hopkins University, Department of Health, Behavior and Society, Bloomberg School of Public Health<br>van Sluijs, Esther; University of Cambridge, CEDAR and MRC Epidemiology Unit
**Primary Subject Heading**:	Epidemiology
Secondary Subject Heading:	Public health, Sports and exercise medicine
Keywords:	Childcare environment, anthropometry, Preschool, physical activity, Nutrition < TROPICAL MEDICINE

SCHOLARONE™ Manuscripts

For peer review only

How does the UK childcare energy-balance environment influence 3-to-4-year-olds' anthropometry? A cross-sectional exploration

Authors

Kathryn R. Hesketh[1,2*]

Sara E. Benjamin-Neelon[1,2]

Esther M.F. van Sluijs[1]

[1] Centre for Diet and Activity Research and MRC Epidemiology Unit, University of Cambridge, Cambridge, UK

[2] Department of Health, Behavior and Society, Johns Hopkins Bloomberg School of Public Health, Baltimore, MD, USA

[*] Corresponding author:

Kathryn.hesketh@ucl.ac.uk

krh40@cam.ac.uk

Address: Centre for Diet and Activity Research and MRC Epidemiology Unit, University of Cambridge School of Clinical Medicine, Cambridge, UK

Telephone: 01223 769169

Word count: 4810

Figures: 0

Tables: 3

Key words: Childcare environment; physical activity; nutrition; Preschool; anthropometry

For peer review only - http://bmjopen.bmj.com/site/about/guidelines.xhtml

ABSTRACT

Objectives To assess the association between time spent in care, the childcare energy-balance environment, and preschool-aged children's Body Mass Index z-score (z-BMI), waist-to-height ratio (WHR), and sum of skinfolds thickness (SST).

Design Cross-sectional study.

Setting and participants 3-to-4-year-old children were recruited from 30 childcare centres in Cambridgeshire (UK) in 2013.

Main outcome measures Objectively measured height and weight was used to calculate BMI z-score; waist circumference and height were used to generate WHR; subscapular and tricep skinfolds were used to calculate SST. Associations between childcare attendance, the nutrition, physical activity, and overall childcare environment, and three anthropometric outcomes were explored using two-level hierarchical regression models, adjusting for demographic and family-based confounders.

Results Valid data were available for 196 children (49% female). Time spent in care, the nutrition, physical activity, and overall childcare environment were not associated with children's z-BMI score, WHR, and SST.

Conclusions Childcare environment and level of attendance were not associated with UK preschool-aged children's anthropometry. The childcare environment has been central to intervention efforts to prevent/ reduce early childhood obesity, yet other factors, including child-, family-, wider environmental, and policy-level factors warrant substantial attention when considering obesity prevention strategies for young children.

For peer review only - http://bmjopen.bmj.com/site/about/guidelines.xhtml

STRENGTHS AND LIMITATIONS OF THIS STUDY

- We used objective measures of UK preschool-aged children's anthropometric indices to provide novel information about how they are associated with childcare attendance and environment.
- This was a relatively small UK childcare-based sample, but our outcomes and exposures were normally distributed, providing sufficient heterogeneity to explore our research questions.
- We used hierarchical regression analysis to take account of study design and potential clustering at the centre level.
- We also adjusted for a number of family-level factors known to be related to children's anthropometry.
- We did not have a measure of each child's birth weight to account for children's individual growth trajectories, nor previous childcare attendance to assess prospective associations.

INTRODUCTION

In 2010, 43 million preschool-aged children worldwide were estimated to be overweight or obese, with a further 92 million at risk of overweight/obesity.[1] Although levels appear to be stabilizing,[2] obesity in childhood is associated with a range of unfavourable outcomes including type 2 diabetes, hyperlipidaemia, and psychosocial problems.[3]

Obesity is often described as an imbalance between energy intake (food consumption) and energy expenditure (physical activity) resulting in excess weight gain over time.[4] As early childhood is a period of rapid growth and development, it represents a key time for establishing healthy energy balance related behaviours (EBRBs), which include physical

For peer review only - http://bmjopen.bmj.com/site/about/guidelines.xhtml
For peer review only

activity, sedentary and dietary behaviours. While parents provide the majority of care for children before they enter school, children now spend increasing amounts of time in non-parental care prior to starting formal education.[5]

In 2014, the average enrolment rate of 3-to-5-year-olds in (pre-school) educational programmes across Organisation for Economic Co-operation and Development (OECD) countries was 80%.[5] In the UK, 3-to-4-year-old children have been entitled to 15 hours of free childcare (38 weeks per year) since 2010,[6] regardless of parental employment, and as of 2017, 3-to-4-year-olds of working parents may be eligible for up to 30 free hours per week.[7] Consequently, in 2017, 95% of UK 3-to-4-year-olds were enrolled in formal care,[8] attending for 21.7 hours per week on average.[9] Preschool-aged children, and particularly those who spend large amounts of time in formal care each week, therefore rely on childcare centres to provide a significant proportion of their food and physical activity opportunities. Elements such as staff education and training, staff behaviour on the playground, lower playground density (less children per square meter), the presence of vegetation and portable toys, and open play areas appear to be positively related to children's physical activity.[10] Likewise, childcare centres and specifically, individual childcare provider practices, appear to be associated with positive dietary behaviours in young children.[11]

Given the influence of the childcare environment on children's EBRBs, and the importance of EBRBs for children maintaining a healthy weight, it follows that time spent in childcare is likely to influence children's weight status. Indeed a number of studies assessing children during infancy and preschool suggest that children attending formal care (vs. those not in formal care) are more likely to be overweight/ obese,[12,13] with both nutrition and physical activity environments contributing.[14] Recently, three systematic reviews[15–17] have been

For peer review only - http://bmjopen.bmj.com/site/about/guidelines.xhtml

conducted to evaluate the link between childcare attendance and obesity-related outcomes, assessing a combined total of 31 papers from North America (n=19), Europe (n=9), and Asia (n=3). Despite the heterogeneous nature of childcare definitions, childcare attendance appears to be associated with increased risk of overweight/obesity,[15–17] with informal care (i.e. relative or non-relative) most commonly found to be associated with increased weight.[15,17] Several studies did however report a decreased risk or no association between informal or centre-based childcare and children's overweight/obesity.[16,17] In general, higher intensity childcare, especially when children attended before one year, increased the overweight/obesity risk, with later (≥3 years old) enrolment in centre-based care associated with lower risk of overweight/obesity compared to earlier enrolment.[17] Lastly, there is some uncertainty as to whether risk differs by socio-demographic factors: children from lower socio-economic backgrounds who spend time in childcare have been shown to have both a greater[18] and lower[19] risk of developing obesity.

Due to the high numbers of children attending formal childcare settings, and therefore the ability to reach large numbers of children in these environments, intervention studies are often conducted in these settings to prevent, halt or reverse obesity during the preschool years. A review by Zhou and colleagues[20] identified 15 such studies: six (of 13) interventions with a dietary component, and eight (of 12) interventions with physical activity component reported improvements in the target EBRB,[20] with seven (47%) reporting subsequent improvements in children's adiposity. Few studies (4/15) assessed long-term efficacy or sustainability (5/15) and the authors also noted heterogeneity across study designs and the interventions delivered, which suggests effects may be limited to specific population subgroups.

For peer review only - http://bmjopen.bmj.com/site/about/guidelines.xhtml

Such findings perhaps reflect differences in policies and practices around nutrition and physical activity across childcare centres (e.g. by level of deprivation[21]) and between countries, which likely influence the extent to which the childcare environment is associated with children's weight indicators. Moreover, uptake of childcare varies between countries,[5] and so too does the amount of time children spend in any form of care,[18] which may also influence the strength of the association between the childcare environment and obesity levels. Finally, it is possible that despite the influence of the childcare environment, parental or family-level factors, which are often targeted simultaneously, may exert a strong or stronger influence on children's weight status.[22]

In England, over 1 in 5 children are overweight or obese by the age of five.[23] Despite high levels of childcare attendance in the United Kingdom (UK), there has been very little research to assess associations between the childcare environment and children's health outcomes. With the publication of the UK Government's Obesity Plan for Action in 2017, strategies to encourage positive health behaviours and weight in preschool-aged children are increasingly centred on the childcare environment.[24] It is therefore timely to determine associations between the UK childcare environment and children's anthropometric indices. In this exploratory study, we therefore sought to assess how the amount of time spent in childcare, and how the nutrition, physical activity and overall childcare environment are associated with anthropometric indicators (z-BMI score; waist-to-height ratio (WHR); sum of skinfold thickness (SST)) in a sample of UK 3-to-4-year-old children, adjusting for a range of family-level explanatory variables. We hypothesised that children attending childcare centres with more supportive physical activity and nutrition environments would have favourable anthropometric indices compared to those attending centres with less supportive environments.

For peer review only - http://bmjopen.bmj.com/site/about/guidelines.xhtml

MATERIALS AND METHODS

Study design and recruitment

Data were from the "Studying Physical Activity in preschool-aged Children and their Environment (SPACE) Study", a cross-sectional childcare-based observational study[25]. The STrengthening the Reporting of OBservational studies in Epidemiology (STROBE) protocol was followed in the conduct and dissemination of this observational study. Recruitment and data collection took place in January-July 2013; detailed information is available elsewhere.[25] Briefly, a list of preschool (state-run education) and nursery (privately-run) 'childcare centres' in Cambridge were obtained from the Ofsted government website[26] and stratified by type (preschool/ nursery) and tertile of index of multiple deprivation (IMD; an area-level measure of deprivation[27]). Preschools and nurseries, but not home/ family-based children centres, were purposively recruited as funding, the built environment and care provided tend to differ by type.[25] Within these six strata, 88 childcare centres were approached at random and invited to participate in writing; 30 (34%) centre managers consented to participate (n=15 preschool and n=15 nursery).

Participants

The parents of all children aged 3-4 years (n=602) attending consenting centres were sent a study invitation pack, and requested to return written consent to the childcare centre. Eligible children were: aged 3 or 4 years; free from physical disability; attended the centre for at least nine hours per week (to ensure children spent >50 % of their government-paid allocation at that particular centre); and registered to attend the childcare centre on the designated measurement day. At least five children per centre with valid written consent (by a parent/legal guardian) were required to ensure sufficient analytical power. Children provided

For peer review only - http://bmjopen.bmj.com/site/about/guidelines.xhtml

verbal assent prior to measurement. The University of Cambridge Psychology Ethics Committee provided ethical approval for the study (Pre.2012.68).

Patient and Public Involvement

Participants were not involved in the development of the study design, research questions or outcome measures, but results were disseminated to all participants and participating childcare centres.

Data collection

Child Anthropometry and Demographic data

At each centre visit, one of three trained researchers recorded each child's sex; measured height to the nearest 0.1cm using a Leicester stadiometer; and weight to the nearest 0.1kg using Seca digital scales in light indoor clothes with shoes and socks removed. Measures of weight and height were conducted once as these are highly reproducible with limited variability. Abdominal waist circumference was measured to the nearest 0.5cm at the midpoint between the lower costal margin and the level of the anterior superior iliac crests. Up to three measures were taken (if there was a discrepancy of 1cm in the first two measures) using a Seca non-stretch tape measure next to the child's skin. Subscapular and tricep skinfolds were measured on the child's right side using a Holtain caliper (Holtain Ltd, United Kingdom) according to standard protocol.[28] If the first two measurements at either site were >0.2mm apart, a third measurement was taken and an average calculated. Compared to a 'gold standard' trainer, researcher mean differences in measurement was 0.1cm and 0.25-0.3mm for waist and skinfolds respectively. Equipment was calibrated prior to commencing data collection, at the mid-way point and on completion of the study.

For peer review only - http://bmjopen.bmj.com/site/about/guidelines.xhtml

Following anthropometric measurement, each child was allocated a study pack containing a parental questionnaire, which care providers disseminated to parents. The parental questionnaire, based on the previously validated questionnaire,[29] assessed demographic factors relating to the study participant; their general health and common health behaviours; childcare attendance; other children in the home; family socio-demographics; parental occupational and leisure physical activity; parental height and weight; and parental beliefs, barriers and attitudes towards physical activity and nutrition. Parents were asked to return the questionnaire to their child's childcare centre one week later.

Assessment of Childcare Environment

A trained researcher assessed the physical activity and nutrition environment of each centre using the Environment and Policy Assessment and Observation (EPAO) instrument. Observation began when the first child arrived in the morning and continued until the end of the day, when the last child left,[30] as the EPAO protocol requires a minimum of one full day to be spent observing all activities in the designated childcare classroom. Scoring is composed of two eight-item subscales for physical activity and nutrition. The physical activity subscales comprise: Active Opportunities, Sedentary Opportunities, Sedentary Environment, Portable Play Environment, Fixed Play Environment, Staff Physical Activity Behaviours, Physical Activity Training and Education, and Physical Activity Policy. Nutrition subscales comprise: Servings of fruits and vegetables; Whole grains; High-sugar/high-fat foods; Beverages; Staff nutrition behaviours; Nutrition environment; Nutrition training and education; and Nutrition policy.

As this tool was developed in the USA, a number of small amendments to the protocol and data collection template were made to ensure the tool was suitable in the UK context. This

For peer review only - http://bmjopen.bmj.com/site/about/guidelines.xhtml

was done in consultation with the original development team to ensure the measure appropriately captured the physical activity and nutrition environments in UK childcare centres. These amendments did not change how the EPAO subscales for physical activity were scored; where preschools did not serve food, we used averages of available variables to derive the nutrition subscale scores.

Variable derivation

Outcome measures

Objectively measured height and weight were used to calculate children's BMI (weight[Kg]/ height^2[m]). This, combined with child's sex and age in months at measurement (calculated from parental reported date of child's birth), was used to derive a continuous BMI z-score, based on the British 1990 (UK90) growth reference charts.[31] International Obesity Task Force classifications were used to categorise children as normal weight, overweight or obese.[32] Waist-to-height ratio (WHR) (waist [cm]/ height [cm]) was derived to assess central obesity, and the sum of subscapular and tricep skinfold thicknesses (SST) was derived as an indicator of subcutaneous fat.[33]

Exposure variables

Parents were asked to provide information about their child's usual weekly childcare attendance using a specifically designed question:[25] "In a usual week, when does your child attend childcare? *Please only include care for your child taking part in SPACE and include regular formal and informal care (e.g. grandparents, friends etc.).*" Parents responded using free text, which we subsequently coded to derive the total number of reported hours children attended *formal* childcare during a usual week for analyses. Indicators of the overall childcare, nutrition, and physical activity environments using EPAO

For peer review only - http://bmjopen.bmj.com/site/about/guidelines.xhtml

(sub-scale) scores were also generated. According to standard EPAO scoring procedure, responses to questions across eight physical activity, and up to eight nutrition sub-domains were summed to a possible maximum of 20 points per domain (where each question was worth 0-2 points). The 'Physical Activity' domain score was derived using an average of the eight subscale domain scores for all centres. The 'Nutrition' domain score was calculated using an average of six **or** eight subdomain scores, depending on meals served. Many UK childcare centres do not serve all meals (i.e. some only serve snacks/ require children to bring packed lunch, and do not provide lunch and 'tea' (at ~4pm)). Two nutrition subscale scores (i.e. Whole grains; High-sugar/high-fat foods) could therefore not be calculated for 21 centres: for these an average of the relevant six subdomain scores was calculated. An overall 'EPAO score' was derived by averaging the eight physical activity, and six or eight nutrition subdomain scores.

Additional confounding variables

A range of potential explanatory variables relating to the child's family environment were derived using data collected from a parental questionnaire, including child ethnicity (White British; White European; Other); maternal educational attainment (General Certificate of Secondary Education (GCSEs); A Levels; National Vocational Qualification (NVQ)/ Diploma; University degree; Higher degree); maternal self-report BMI; and hours per week mothers worked (Not employed; ≤20 hours; 21-35 hours; >35 hours). Where available, the latter three variables were also derived for fathers (with hours per week fathers worked categorised based on distribution as: <40 hours; 40-42 hours; >42 hours).

Statistical Analyses

For peer review only - http://bmjopen.bmj.com/site/about/guidelines.xhtml

All analyses were conducted using STATA 14/SE. We calculated descriptive statistics for included participants, and compared them to those excluded from analyses (i.e. those without valid anthropometric data) using independent t-tests and Pearson's chi^2.

For each of our three continuous outcome variables (z-BMI score, WHR, SST), a series of two-level mixed-effects linear regression analyses (level 1: child; level 2: childcare centre) were conducted to explore how weekly childcare attendance and practices relating to energy-balance behaviours in the childcare environment were related to children's anthropometric indices. Given children's age in months and sex are already taken into account when z-BMI score is derived, all WHR and SST analyses were adjusted for these variables. As children in the SPACE study were recruited or 'clustered' at the centre level, multi-level regression analyses were used to allow for both within- and between-centre variation in anthropometric outcomes.[25,34] First, univariate analyses were conducted to assess how a) weekly childcare attendance (in hours), and b) the childcare environment relating to nutrition and physical activity, were associated with each of the three anthropometric indices. Multivariable analyses were then run, adjusting for family-level confounding variables. One centre contributed two classes, which were treated as separate centres in analyses: although the classes shared policy documents, each ran as a completely independent entity, with different staff, rooms, children and outside spaces, and did not share catering facilities (hot meals were not provided).

Significant within-centre differences across the three outcome measures were identified. For example, 96% of the variation in z-BMI score was explained by within-centre (i.e. child-level) differences and only 4% of variation was explained by between-centre (i.e. childcare-level) differences [within-centre variation WHR: 73%; STT: 85%]. To further explore these

For peer review only - http://bmjopen.bmj.com/site/about/guidelines.xhtml

differences, *post hoc* analyses were conducted to determine whether child-level socio-economic status (measured using maternal educational attainment) moderated the association between time spent in the childcare environment and each outcome. Based on previous evidence, we hypothesised that the relationship between a more favourable childcare environment and normal weight would be stronger (i.e. the gradient would be steeper) for children in lower income families. This was grounded on the assumption that favourable childcare environments would buffer the association between potentially negative home environments and overweight/ obesity in children from poorer socio-economic backgrounds, but that the childcare environment may be less important for children from higher income homes. Also, as fewer children provided SST data than z-BMI score and WHR, sensitivity analyses were conducted to limit our analyses to children providing complete case valid data for all three outcomes (n=151).

RESULTS

Participant Characteristics

Of the 245 children who assented to have anthropometric measurements taken, valid anthropometric, observational, and questionnaire data were available for 196 children from 30 centres (Table 1). Childcare centres have been described previously;[35] those centres who participated did not differ in terms of area-level socio-economic characteristics from those who declined to participate.[25] Children who provided complete case data (n=196) did not differ from those excluded by child's sex, age, weight status or ethnicity but were more likely to have higher educated mothers (higher degree: 38.3% vs. 22.2%, p=0.02).

For peer review only - http://bmjopen.bmj.com/site/about/guidelines.xhtml

Table 1 Descriptive characteristics of children included in analyses (SPACE study, 2013, N=196)

	Boys	Girls
**Child Characteristics**		
N (%)	100 (51)	96 (49)
Age (in months)	47.4 (5.2)	47.8 (4.9)
Ethnicity (N(%))		
White British	74 (74.0)	74 (77.1)
White other	9 (9.1)	9 (9.4)
Other/ mixed ethnicity	17 (17.0)	13 (13.5)
BMI z-score	0.52 (0.93)	0.36 (1.06)
Weight category[a] (N(%))		
Normal	82 (82.0)	79 (82.3)
Overweight/obese	18 (18.0)	17 (17.7)
Waist/ Height ratio	0.49 (0.1)	0.49 (0.1)
Centrally obese (N(%))	35 (46.7)	28 (40.6)
Sum of skinfolds[b] (cm)	14.5 (3.1)	16.0 (3.6)
Average weekly hours in childcare	23.3 (12.2)	21.8 (12.1)
**Maternal Characteristics**		
Age (in years)	37.6 (5.0)	37.4 (5.5)
BMI (in kg/m^2)	23.9 (4.2)	24.2 (4.7)
Education (N(%))		
GCSE/A-levels	25 (25.0)	34 (35.4)
Degree	29 (29.0)	33 (34.4)
Higher degree	46 (46.0)	29 (30.2)
Hours worked per week[c] (N(%))		
Not employed	26 (27.4)	24 (25.8)
<20 hours	19 (20.0)	14 (15.1)
21-35 hours	28 (29.5)	31 (33.3)
>35 hours	22 (23.2)	24 (25.8)
**Paternal Characteristics[e]**		
Age (in years)	39.2 (5.6)	39.4 (8.0)
BMI (in kg/m^2)	25.2 (3.5)	25.9 (3.2)
Paternal education (N(%))		
GCSE/A-levels	23 (23)	20 (24.1)
Degree	24 (24)	27 (32.5)
Higher degree	43 (43)	36 (43.4)
Hours worked per week[c] (N(%))		
<40 hours	26 (29.8)	30 (35.3)
40-42 hours	30 (35.6)	30 (36.6)
>42 hours	30 (34.5)	22 (26.8)

All values mean(SD) unless stated otherwise; BMI: Body mass index; a: Weight category derived using the International Task Force on Obesity cut points; GCSE: General Certificate of Secondary Education; A-levels: Advanced Levels; b: n=80 boys and n=71 girls; c: categorized based on distribution; c: maternal employment: n=95 for boys, n=93 for girls; d: paternal variables available for n=169-173 children depending on variable

For peer review only - http://bmjopen.bmj.com/site/about/guidelines.xhtml

Childcare Environment

Domain subscale and total scores for the EPAO are shown in Table 2. Across childcare centres, the mean total EPAO score (including eight physical activity and up to eight nutrition domains) was 11.2 (SD: 1.0, range: 8.5 - 13.5), with higher scores signifying more supportive environments. The average physical activity subscale score was 10.8 (1.5, 7.4-13.8) and average nutrition subscale score was 11.7 (1.6, 8.8-14.3). Overall, nutrition scores indicated good provision of fruit and vegetables, limited servings of high-fat/high-sugar foods, but poor provision of wholegrains. For physical activity, centres generally scored well for active opportunities and staff physical activity behaviours, but staff training and education in physical activity, and physical activity policies, were largely lacking.

For peer review only

Table 2 EPAO average domain subscale and total scores in the SPACE study

Nutrition sub-domains[a]	Mean (SD)	Range	Physical Activity sub-domains[b]	Mean (SD)	Range
Servings: fruits and vegetables	13.4 (4.1)	6.7-20.0	Active Opportunities	15.3 (3.8)	6.7 – 20.0
Servings: Whole grains[c]	3.3 (3.3)	0-10.0	Sedentary Opportunities	11.7 (4.5)	6.7 – 20.0
Servings: High-sugar/high-fat foods[d,e]	14.5 (0.9)	14.0 –16.0	Sedentary Environment	12.7 (4.1)	6.7 – 20.0
Servings: Beverages	9.7 (2.4)	5.0 - 16.3	Portable Play Environment	10.7 (3.1)	5.7 - 17.1
Staff nutrition behaviours	10.1 (2.8)	3.3 -15.0	Fixed Play Environment	11.8 (2.8)	7.5 - 16.3
Nutrition environment	15.4 (3.0)	6.7 – 20.0	Staff PA Behaviours	14.3 (2.5)	8.0 – 20.0
Nutrition training and education	10.6 (3.1)	4.0 – 18.0	PA Training and Education	4.8 (4.0)	0 - 15.0
Nutrition policy	12.6 (6.2)	6.7 – 20.0	PA Policy	7.0 (6.8)	0 - 20.0
**Total Domain Score[b]**					
Average Nutrition domain score[f]	11.7 (1.6)	8.8 - 14.3	Average Physical Activity domain score[g]	10.8 (1.5)	7.4 - 13.8
**Total EPAO Score[b]**					
Average total EPAO score[h]	11.2 (1.0)	8.5 - 13.5			

EPAO: Environment and Policy Assessment and Observation tool; a: n=30 unless stated; b: n=30; c: centres n=9; d: centres n=8; e: reverse coded such that higher score means favourable or lower provision of high-sugar, high-fat foods; f: average of up to 8 subdomain scores; g: includes 8 subdomain scores; h: average of up to 8 nutrition and 8 physical activity domain scores

For peer review only - http://bmjopen.bmj.com/site/about/guidelines.xhtml

Association between childcare environment and children's anthropometric indices

In unadjusted analyses, there was no significant association between the number of hours a child spent in care or energy-balance childcare practices and preschool-aged children's anthropometric indices (Table 3). These findings remained unchanged after adjusting for family-level variables; as expected, several family-level variables were independently associated with the outcomes of interest in adjusted models (see supplementary Table S1).

We found no significant interactions in *post hoc* analyses, designed to determine whether child-level socio-economic status (measured using maternal educational attainment) moderated the association between time spent in the childcare environment and our three outcome measures (data not shown). In sensitivity analyses, including only children who provided all three outcome measures (n=151) did not significantly influence our findings (data not shown).

For peer review only

For peer review only - http://bmjopen.bmj.com/site/about/guidelines.xhtml

Table 3 Associations between childcare environment and preschool-aged children's anthropometric indices in the SPACE study

	Exposure measures β [95% CI]							
	Weekly hours in care		EPAO PA score		EPAO Nutrition score		EPAO total score	
**Continuous Outcomes**	Unadjusted	Adjusted[a]	Unadjusted	Adjusted[a]	Unadjusted	Adjusted[a]	Unadjusted	Adjusted[a]
BMI z-score[b]	-0.00	-0.01	-0.02	-0.01	0.01	-0.01	-0.00	-0.00
	[-0.02,0.01]	[-0.02,0.00]	[-0.12,0.09]	[-0.12,0.11]	[-0.09,0.10]	[-0.11,0.09]	[-0.01,0.01]	[-0.01,0.01]
Waist to height ratio[b,c]	-0.00	-0.00	0.01	0.00	-0.00	-0.00	0.00	0.00
	[-0.00,0.00]	[-0.00,0.00]	[-0.01,0.02]	[-0.01,0.02]	[-0.01,0.01]	[-0.01,0.01]	[-0.00,0.00]	[-0.00,0.00]
Sum of skinfold[c,d]	0.02	0.03	0.03	0.18	-0.03	-0.18	0.00	0.00
	[-0.02,0.07]	[-0.01,0.08]	[-0.44,0.50]	[-0.30,0.67]	[-0.45,0.40]	[-0.61,0.26]	[-0.05,0.05]	[-0.05,0.05]

EPAO: Environment and Policy Assessment and Observation tool; a: model adjusted for child ethnicity, maternal BMI, maternal educational attainment and maternal working hours; b: n=196; c: model also includes child sex and age in months; d: n=144

For peer review only

For peer review only - http://bmjopen.bmj.com/site/about/guidelines.xhtml

DISCUSSION

We found that childcare attendance and energy-balance practices in the childcare environment do not appear to be associated with UK preschool-aged children's anthropometric indices. However, as shown previously, family-level factors were independently associated with children's z-BMI score. Children spend increasing amounts of time in formal childcare in the UK and the childcare environment is frequently the focus of intervention efforts to prevent or reduce early childhood obesity worldwide.[24] Childcare centres in the UK adhere to a statutory Early Years Foundation Stage (EYFS) framework, operate 'free-flow' policies were children generally choose their own activities with few provider-led activities, and by law must ensure all food and drink provided is properly prepared, wholesome and nutritious.[36] Therefore, this relatively standardised level of care may mean that UK childcare environments exert a smaller influence on children's EBRBs and health. Together, this suggests that child-, family-, environmental-, and policy-level factors warrant significant further attention in obesity prevention strategies for young children.

How this work compares to previous studies

Cross-sectionally, we did not identify any associations between time spent in care and children's anthropometry. This is in accordance with previous studies conducted in low-income Latino 4-year-olds[37] and 2-5-year olds in Australia[38]. Interestingly, the latter study identified maternal education as the sole significant predictor of child's weight status.[38] Although consideration of family level factors do not impact the conclusions drawn here (i.e. no association was found before or after adjustment), several were independently associated with BMI z-score, significantly attenuating the (non-significant) relationship between exposure and outcome. This confirms previous findings that family

For peer review only - http://bmjopen.bmj.com/site/about/guidelines.xhtml

level factors may influential to children's weight status, regardless of childcare attendance.[22]

Within the SPACE study, there was relatively wide heterogeneity in design and layout of childcare centres. Yet EPAO scores in this study showed comparatively little variation across childcare centres and were similar to those seen previously in US,[39] Dutch,[35] and Canadian[40] studies which suggests there were no obvious differences between these UK and other childcare environments. However, across and between countries differences may exist in how 'healthy' childcare environments are, and there may also be variation across physical activity and nutrition environments within the same childcare environment (i.e. good nutrition, poor physical activity and vice versa). Taken together, the definition of a 'healthy' environment is likely to be relatively heterogeneous, potentially preventing identification of consistent associations.

It could be hypothesised that childcare in earlier childhood (i.e. infancy), rather than during the preschool-period (as assessed here), is more important for the development of children's adiposity. Indeed, work conducted in prospective cohorts suggests that early childcare attendance may be associated with children's weight status: Black and colleagues noted that early informal care (before 3 years) was associated with increased risk of overweight/obesity, and that higher childcare attendance, especially starting before 1 year, also increased overweight/obesity risk.[41] This was confirmed in studies in younger children (aged 1-2 years) from Ireland and The Netherlands. The latter suggest that attending childcare part- or full-time was associated with increases in anthropometric indicators and odds of being overweight compared to those not in childcare prospectively.[42,43] Moreover, cumulative exposure to centre-based care up to the age of 4 appears to be associated with higher odds of overweight/obesity in later childhood, i.e. at 4-10 years of age,[12] in Canadian children. As

For peer review only - http://bmjopen.bmj.com/site/about/guidelines.xhtml
For peer review only

such, these longitudinal associations appear to be dependent on the timing and level of exposure to childcare.[15,41] Greater childcare attendance in very early childhood appears to be associated with childhood obesity, but later childcare attendance does not show such associations.[41] As no data on previous childcare attendance patterns were collected in this study, we were unable to test this hypothesis here.

It is conceivable that bias or residual confounding, in addition to our sample size, limited our ability to find associations; it is also possible that a lack of power (i.e. low β) resulted in a type 2 error, or us failing to find an association where one existed. This said, we were able to detect relatively small associations between the outcome and family level factors (e.g. maternal BMI: β co-efficient=0.05), and had suitable heterogeneity in our exposure measures to do so. Previous studies have identified associations with a range of time-based exposures assessed over longer periods of time (i.e. 10 hours/week increments;[44] each additional 30 days exposure to care[13] during the first year of life) or simply by the type of care children were exposed to (i.e. childcare vs. informal vs. parental care[12,18]).[15] This variation in exposure and outcome measures is therefore also likely to account for differing findings between childcare-related factors and children's anthropometric indices.

This is to our knowledge one of the few studies to go beyond exploration of the association between time spent in care and anthropometry, to assess how elements within the childcare environment (i.e. nutrition and physical activity practices) are related to a children's anthropometric indices. In a previous study using this same sample of children, we found a similar lack of association between the childcare environment and preschool-aged children's physical activity[35] as identified here between the preschool environment and children' anthropometry. This is in contrast to studies in the US[39] and Canada[45] suggesting that

For peer review only - http://bmjopen.bmj.com/site/about/guidelines.xhtml

more favourable physical activity environments are associated with higher physical activity levels in children. It is possible that differences identified here are due in part to a fairly standardised system of care in the early years in the UK mentioned earlier. The EYFS framework [46] is a curriculum against which children's development is judged at age 2 and 5 years (after their first year in primary school). A key tenant of this is that children must "be helped to understand the importance of physical activity, and to make healthy choices in relation to food." (Pg 5;[46]) All food and drink served to children in UK early years settings must by law be properly prepared, wholesome and nutritious, with fresh drinking water available to children at all times.[36] UK childcare centres additionally operate a 'free-flow' policy were children may choose from a range of inside and outside activities for the majority of the day, regardless of weather conditions. With a standardised level of provision, the childcare environment in the UK may exert only a small influence on children's EBRBs and health outcomes. Yet with a core mandate to promote healthy EBRBs, and with high levels of children attending childcare, childcare settings in the UK appear well placed to ensure all children receive the best start in life, irrespective of socio-demographic factors.

To date, much of the research in this area has focused on the formal childcare environment. Strategies to encourage positive health behaviours and weight in preschool-aged children are therefore increasingly centred on the childcare environment, particularly in the UK.[24] However, very little research has been conducted in the UK childcare setting to determine whether such interventions are necessary or likely to succeed. Indeed, this and previous research[35] suggests that the formal childcare environment in the UK does not appear to be associated with preschool-aged children's anthropometric and physical activity outcomes. Other types of care (e.g. family child care homes/ childminder; grandparents) however also deserve attention. For example in Canada, preschool-aged children in full day kindergarten

For peer review only - http://bmjopen.bmj.com/site/about/guidelines.xhtml

(providing instructive programmes for preschoolers) accumulated significantly more MVPA than those in centres (providing developmental programmes for a range of ages) or home-base care,[45] whereas in another study, EPAO nutrition subscales in centre-based care were shown to be more supportive than those in home-based care.[40] Given UK preschool-aged children spent an average of 15 hours in informal care in 2017,[9] more research is needed into the potential influences of these informal environments. This said, areas for improvement within the formal childcare environment likely still exist, with a more targeted approach, focusing on specific areas within the childcare environment (e.g. promoting healthy dietary provision[21], improving physical activity policies and staff training), still potentially benefitting children's health behaviours.

Finally, the children's family and wider environments should not be overlooked. Although the family is suggested to be central to health promotion in young children, both more proximal family and external factors (e.g. in the community and wider environment) combine to shape a child's health behaviours.[47] Indeed, parents and childcare providers often cite each other as important custodians of preschool-aged children's EBRBs, suggesting both should work synergistically to encourage positive health and habit formation.[48]

Strengths and limitations

We used objective measures of UK preschool-aged children's anthropometric indices to provide novel information about how attendance and the childcare environment are associated with children's anthropometric indices. Although this was a relatively small UK childcare-based sample, both our outcomes and exposures were normally distributed, and provided us with sufficient heterogeneity to explore our research questions. Although derivation of one of the exposure measures (i.e. the Nutrition domain score) differed from

For peer review only - http://bmjopen.bmj.com/site/about/guidelines.xhtml

previous studies, limiting the number of sub-scale scores used (to 6 rather than taking an average of up to 8) did not influence our findings. By using hierarchical regression analysis, we were also able to take account of study design and potential clustering at the centre-level, thus increasing our power to detect (small) significant differences in our outcomes. We also adjusted for a number of family-level factors known to be related to children's anthropometry but we did not have a measure of each child's birth-weight to account for children's individual growth trajectories. As with several studies in this area,[15] this work was based on childcare-based sample. We were therefore unable to assess the influence of no or only informal care, in addition to centre-based care, on children's weight-related outcomes.

Children were drawn from childcare centres recruited from the top tertile of Index of Multiple Deprivation (IMD) scores in England.[25] As there were no differences in IMD scores of the centres that did and did not participate in the SPACE study,[35] children included here are likely representative of the wider eligible population. Children's individual socio-economic circumstances (i.e. maternal educational attainment) varied within this sample, which is important as children from both lower and higher socio-economic backgrounds may be at risk of higher BMI.[18,19,23] It is not therefore not clear how generalizable our findings are. However, we did not see evidence of a moderating effect of children's socio-economic circumstances on the exposure-outcome relationship, suggesting that the association may be consistent across socio-economic strata. Just under one-fifth (18%) of children were classified as overweight or obese, which is slightly below the national UK average for 5-year old children (22.5% in 2013/14).[23] Children were predominantly White (British/ European) (85%), which is in line with the UK average (86% in 2011).[49] Work is however required to determine whether similar findings are apparent in differing minority populations across the UK.

For peer review only - http://bmjopen.bmj.com/site/about/guidelines.xhtml

CONCLUSIONS

We found no significant association between childcare attendance, or the nutrition and physical activity childcare environment, and children's anthropometric outcomes, suggesting the UK childcare environment had little influence on children's weight status in our study. In contrast, family-level factors were associated. Although childcare-based interventions are increasingly the focus for promoting healthy weight and EBRBs in preschool-aged children, they tend to show small effects that are not sustained over time. Looking to other areas of a child's life, specifically family-level factors and those in a child's wider environment, either as an adjunct or alternative to centre-based interventions, should become a focus. Considering how a broader range of potential influences may interact to contribute to children's health will be key in successfully promoting healthy weight in preschool-aged children.

For peer review only

ACKNOWLEDGMENTS

We thank all children and their parents who participated in the SPACE study. In addition, we thank members of the MRC Epidemiology Unit for their assistance: to members of the field team who conducted data collection, to Kate Westgate and Stefanie Hollidge from the physical activity technical team for the accelerometer data processing, and to Dr Emanuella De Lucia Rolfe and Richard Powell for processing the anthropometric data.

CONTRIBUTORSHIP

KH and EvS conceived of the SPACE study. KH collected the data, conducted the analyses and drafted the manuscript. KH, SBN and EvS interpreted the results and critically revised the manuscript. All authors approved the final manuscript and agree to be accountable for all aspects of the work.

FUNDING

This work was conducted by the Medical Research Council [Unit Programme number MC_UU_12015/7] and the Centre for Diet and Activity Research (CEDAR), a UKCRC Public Health Research Centre of Excellence. Funding from the British Heart Foundation, Cancer Research UK, Economic and Social Research Council, Medical Research Council, the National Institute for Health Research, and the Wellcome Trust, under the auspices of the UK Clinical Research Collaboration, is gratefully acknowledged (CEDAR grant numbers: ES/G007462/1; 087636/Z/08/Z; MR/K023187/1). KH is funded by the Wellcome Trust (107337/Z/15/Z). SBN is funded by NIH and the Robert Wood Johnson Foundation.

COMPETING INTERESTS

All authors have completed the ICMJE uniform disclosure form at http://www.icmje.org/coi_disclosure.pdf (available on request from the corresponding author) and declare that (1) they have support from a number of grants outlined above; (2) all authors have no relationships with any organisations that might have an interest in the submitted work in the previous 3 years; (3) their spouses, partners or children have no financial relationships that may be relevant to the submitted work and (4) have no non-financial interests that may be relevant to the submitted work.

For peer review only

For peer review only - http://bmjopen.bmj.com/site/about/guidelines.xhtml

ETHICS APPROVAL

Ethical approval was granted by The University of Cambridge Psychology Ethics Committee for the study (Pre.2012.68).

DATA SHARING STATEMENT

Unpublished data regarding children's health behaviours, socio-economic circumstances and parental behaviours are available for the SPACE study. The anonymised data set and statistical code used for analyses are available from the corresponding author on request. Model parameters are outlined in the 'Methods' section. Participants gave assent and parents provided informed consent for study participation, but the presented data are anonymised and risk of identification is very low.

For peer review only

REFERENCES

1 de Onis M, Blossner M, Borghi E. Global prevalence and trends of overweight and obesity among preschool children. *Am J Clin Nutr* 2010;92:1257–64.

2 Rokholm B, Baker J, Sørensen T. The levelling off of the obesity epidemic since the year 1999 - a review of evidence and perspectives. *Obes Rev* 2010;11:835–46.

3 Lakshman R, Elks CE, Ong KK. Childhood Obesity. *Circulation* 2012;126:1770–9.

4 World Health Organisation. Obesity and overweight. WHO Fact sheet N°311. 2013. http://www.who.int/mediacentre/factsheets/fs311/en/ (accessed 3 Mar 2017).

5 OECD. Equity and Quality in Education: Supporting Disadvantaged Students and Schools. OECD. 2012. http://dx.doi.org/10.1787/9789264130852-en (accessed 3 Feb 2015).

6 Office for Standards in Education (Ofsted). Early Years and Childcare. www.gov.uk. 2016. http://www.ofsted.gov.uk/early-years-and-childcare (accessed 30 Dec 2013).

7 UK Government. Help paying for childcare. 2018. https://www.gov.uk/help-with-childcare-costs/free-childcare-and-education-for-2-to-4-year-olds (accessed 2 Mar 2018).

8 UK Government. Education provision: children under 5 years of age, January 2017. Childcare and Early Years. 2017. https://www.gov.uk/government/statistics/education-provision-children-under-5-years-of-age-january-2017 (accessed 28 Feb 2018).

9 Department for Education. Childcare and early years survey of parents: 2017. https://www.gov.uk/government/uploads/system/uploads/attachment_data/file/669857/SFR73_2017_Text.pdf (accessed 9 Mar 2018).

10 Trost SG, Ward DS, Senso M. Effects of child care policy and environment on physical activity. *Med Sci Sport Exerc* 2010;42:520–5.

11 Gubbels JS, Gerards SMPL, Kremers SPJ. Use of food practices by childcare staff and

For peer review only - http://bmjopen.bmj.com/site/about/guidelines.xhtml

the association with dietary intake of children at childcare. *Nutrients* 2015;7:2161–75.

12 Geoffroy M-C, Power C, Touchette E, et al. Childcare and overweight or obesity over 10 years of follow-up. *J Pediatr* 2013;162:753–758.e1.

13 Benjamin Neelon SE, Schou Andersen C, Schmidt Morgen C, et al. Early child care and obesity at 12 months of age in the Danish National Birth Cohort. *Int J Obes* 2015;39:33–8.

14 Sisson SB, Li J, Stoner JA, et al. Obesogenic environments in tribally-affiliated childcare centers and corresponding obesity rates in preschool children. *Prev Med Reports* 2016;3:151–8.

15 Alberdi G, McNamara AE, Lindsay KL, et al. The association between childcare and risk of childhood overweight and obesity in children aged 5 years and under: a systematic review. *Eur J Pediatr* 2016;175:1277–94.

16 Costa S, Adams J, Gonzalez-nahm S, et al. Childcare in Infancy and Later Obesity : a Narrative Review of Longitudinal Studies. *Curr Pediatr Reports* 2017;5:118–31.

17 Black L, Kearney PM. The association between childcare arrangements and risk of overweight and obesity in childhood: a systematic review. *Obes Rev* 2017;18(10):1170-1190 18

18 Pearce A, Li L, Abbas J, et al. Is childcare associated with the risk of overweight and obesity in the early years? Findings from the UK Millennium Cohort Study. *Int J Obes* 2010;34:1160–8.

19 Nazarov Z, Rendall M. Differences by mother's education in the effect of childcare on child obesity. *Econ Lett* 2014;124:286–9.

20 Zhou YE, Emerson JS, Levine RS, et al. Childhood obesity prevention interventions in childcare settings: Systematic review of randomized and nonrandomized controlled trials. *Am J Heal Promot* 2014;28:92–104.

For peer review only - http://bmjopen.bmj.com/site/about/guidelines.xhtml

21 Neelon SEB, Burgoine T, Hesketh KR, et al. Nutrition practices of nurseries in England: Comparison with national guidelines. *Appetite* 2015;85:22-9.

22 Hawkins SS, Cole TJ, Law C. An ecological systems approach to examining risk factors for early childhood overweight: findings from the UK Millennium Cohort Study. *J Epidemiol Community Health* 2009;63:147–55.

23 NHS Digital. National Child Measurement Programme - England, 2016-17. https://digital.nhs.uk/catalogue/PUB30113 (accessed 4 Nov 2017).

24 Department of Health. Childhood Obesity: A Plan for Action. London, UK: 2016. https://www.gov.uk/government/uploads/system/uploads/attachment_data/file/546588/Childhood_obesity_2016__2__acc.pdf (accessed 4 Nov 2017).

25 Hesketh KR, Griffin SJ, van Sluijs EMF. UK Preschool-aged children's physical activity levels in childcare and at home: a cross-sectional exploration. *Int J Behav Nutr Phys Act* 2015;12:123.

26 Office for Standards in Education (Ofsted). Types of early years and childcare Ofsted inspects. 2013. http://www.ofsted.gov.uk/early-years-and-childcare (accessed 29 Nov 2014).

27 UK Government. English Indices of Deprivation 2010. https://www.gov.uk/government/statistics/english-indices-of-deprivation-2010 (accessed 4 Jan 2013).

28 MRC Epidemiology Unit, University of Cambridge. Simple measurements - skinfolds. DAPA Toolkit. http://dapa-toolkit.mrc.ac.uk/anthropometry/objective-methods/simple-measures-skinfolds (accessed 4 Feb 2018).

29 McMinn AM, van Sluijs EM, Harvey NC, et al. Validation of a maternal questionnaire on correlates of physical activity in preschool children. *Int J Behav Nutr Phys Act* 2009;6:81.

For peer review only - http://bmjopen.bmj.com/site/about/guidelines.xhtml

For peer review only

30 Ammerman AS, Ward DS, Benjamin SE, et al. An Intervention to Promote Healthy weight: Nutrition and Physical Activity Self-Assessment for Child Care (NAP SACC) theory and design. *Prev Chronic Dis* 2007;4:A67.

31 Cole TJ, Freeman J V, Preece MA. Body mass index reference curves for the UK, 1990. *Arch Dis Child* 1995;73:25–9.

32 Cole TJ, Lobstein T. Extended international (IOTF) body mass index cut-offs for thinness, overweight and obesity. *Pediatr Obes* 2012;7:284–94.

33 Freedman DS, Katzmarzyk PT, Dietz WH, et al. Relation of body mass index and skinfold thicknesses to cardiovascular disease risk factors in children : the Bogalusa Heart Study 1 – 4. *Am J Clin Nutr* 2009;90:210–6.

34 Goldstein H. *Multilevel Statistical Models*. 4th ed. Wiley-Blackwell 2010.

35 Hesketh KR, van Sluijs EMF. Features of the UK childcare environment and associations with preschooler's in-care physical activity. *Prev Med Reports* 2016;3:53–7.

36 The Office for Standards in Education Children's Services and Skills (Ofsted). Early years and childcare registration handbook. Manchester, UK: 2017. https://www.gov.uk/government/uploads/system/uploads/attachment_data/file/650103/EY_and_childcare_reg_handbook.pdf (accessed 23 Feb 2018).

37 Zahir N, Heyman MB, Wojcicki JM. No association between childcare and obesity at age 4 in low-income Latino children. *Pediatr Obes* 2012;8:E24-28.

38 Wolfenden L, Hardy LL, Wiggers J, et al. Prevalence and socio-demographic associations of overweight and obesity among children attending child-care services in rural and regional Australia. *Nutr Diet* 2011;68:15–20.

39 Bower JK, Hales DP, Tate DF, et al. The Childcare Environment and Children's Physical Activity. *Am J Prev Med* 2008;34:23–9.

For peer review only - http://bmjopen.bmj.com/site/about/guidelines.xhtml

40 Martyniuk OJ, Vanderloo LM, Irwin JD, et al. Comparing the nutrition environment and practices of home- and centre-based child-care facilities. *Public Health Nutr* 2016;19:575–84.

41 Black L, Matvienko-Sikar K, Kearney PM. The association between childcare arrangements and risk of overweight and obesity in childhood: a systematic review. *Obes Rev* 2017;18:1170–90.

42 Gubbels JS, Kremers SPJ, Stafleu A, et al. Child-care use and the association with body mass index and overweight in children from 7 months to 2 years of age. *Int J Obes* 2010;34:1480–6.

43 Scully H, Alberdi G, Segurado R, et al. Child Care Exposure Influences Childhood Adiposity at 2 Years: Analysis from the ROLO Study. *Child Obes* 2017;13:93–101.

44 Benjamin SE, Rifas-Shiman SL, Taveras EM, et al. Early child care and adiposity at ages 1 and 3 years. *Pediatrics* 2009;124:555–62.

45 Vanderloo LM, Tucker P, Johnson AM, et al. Environmental Influences on Preschoolers' Physical Activity Levels in Various Early-Learning Facilities. *Res Q Exerc Sport* 2015;86:360–70.

46 Department for Education. Early Years Foundation Stage (EYFS). United Kingdom: 2012. http://www.education.gov.uk/aboutdfe/statutory/g00213120/eyfs-statutory-framework (accessed 19 Mar 2013).

47 Christensen P. The health-promoting family: a conceptual framework for future research. *Soc Sci Med* 2004;59:377–87.

48 Hesketh KR, Lakshman R, van Sluijs EMF. Barriers and facilitators to young children's physical activity and sedentary behaviour: a systematic review and synthesis of qualitative literature. *Obes Rev* 2017; 18(9):987-1017

49 Office of National Statistics. Ethnicity and National Identity in England and Wales:

For peer review only - http://bmjopen.bmj.com/site/about/guidelines.xhtml

2011. London, UK: 2012. http://www.ons.gov.uk/peoplepopulationandcommunity/culturalidentity/ethnicity/articles/ethnicityandnationalidentityinenglandandwales/2012-12-11 (accessed 19 Mar 2013).

For peer review only

For peer review only - http://bmjopen.bmj.com/site/about/guidelines.xhtml

Supplementary Table S1: Associations between childcare attendance and environment and BMI z-score (n=196)

	Exposure Measures β [95% CI]							
	Weekly hours in care		EPAO PA score		EPAO Nutrition score		EPAO total score	
	Unadjusted	Adjusted	Unadjusted	Adjusted	Unadjusted	Adjusted	Unadjusted	Adjusted
Constant	0.51 [0.22,0.81]	-0.35 [-1.27,0.56]	0.62 [-0.52,1.77]	-0.50 [-2.02,1.02]	0.36 [-0.82,1.53]	-0.45 [-1.91,1.00]	0.58 [-1.11,2.26]	-0.39 [-2.33,1.56]
**Child factors**								
Ethnicity (ref: white)								
White European		-0.06 [-0.56,0.44]		-0.10 [-0.61,0.40]		-0.10 [-0.61,0.40]		-0.10 [-0.61,0.41]
Other/ mixed		0.09 [-0.30,0.49]		0.05 [-0.35,0.45]		0.05 [-0.35,0.44]		0.05 [-0.34,0.45]
**Maternal Factors**								
Maternal BMI		0.05** [0.02,0.08]		0.05*** [0.02,0.08]		0.05*** [0.02,0.08]		0.05*** [0.02,0.08]
Maternal education (ref: GCSE/ A-levels)								
Degree		-0.49** [-0.83,-0.15]		-0.52** [-0.86,-0.18]		-0.52** [-0.86,-0.18]		-0.52** [-0.86,-0.18]
Higher degree		0.04 [-0.31,0.39]		0.01 [-0.34,0.35]		0.01 [-0.33,0.36]		0.01 [-0.33,0.35]
Maternal working hours (ref: not employed)								
<20 hours		-0.37 [-0.79,0.04]		-0.40 [-0.81,0.02]		-0.40 [-0.81,0.02]		-0.40 [-0.81,0.02]
21-35 hours		-0.01 [-0.38,0.36]		-0.06 [-0.42,0.30]		-0.06 [-0.41,0.30]		-0.05 [-0.41,0.31]
>35 hours		0.20 [-0.21,0.61]		0.09 [-0.29,0.47]		0.09 [-0.29,0.47]		0.09 [-0.29,0.47]

95% confidence intervals in brackets; * $p < 0.05$, ** $p < 0.01$, *** $p < 0.001$

For peer review only - http://bmjopen.bmj.com/site/about/guidelines.xhtml

For peer review only

Supplementary Table S2: Associations between childcare attendance and environment and Waist to Height Ratio (n=188)

	Exposure Measures β [95% CI]							
	Weekly hours in care		EPAO PA score		EPAO Nutrition score		EPAO total score	
	Unadjusted	Adjusted	Unadjusted	Adjusted	Unadjusted	Adjusted	Unadjusted	Adjusted
Constant	0.48*** [0.36,0.60]	0.50*** [0.36,0.65]	0.42*** [0.25,0.59]	0.44*** [0.24,0.64]	0.51*** [0.34,0.67]	0.53*** [0.35,0.71]	0.44*** [0.23,0.65]	0.47*** [0.24,0.71]
**Child factors**								
Sex	0.01 [-0.02,0.03]	0.01 [-0.02,0.03]	0.01 [-0.02,0.03]	0.01 [-0.02,0.03]	0.01 [-0.02,0.03]	0.01 [-0.02,0.03]	0.01 [-0.02,0.03]	0.01 [-0.02,0.03]
Age in months	0.00 [-0.00,0.00]	0.00 [-0.00,0.00]	0.00 [-0.00,0.00]	0.00 [-0.00,0.00]	0.00 [-0.00,0.00]	0.00 [-0.00,0.00]	0.00 [-0.00,0.00]	0.00 [-0.00,0.00]
Ethnicity (ref: white)								
White European		0.01 [-0.04,0.06]		0.00 [-0.05,0.05]		0.01 [-0.04,0.05]		0.00 [-0.04,0.05]
Other/ mixed		0.00 [-0.04,0.04]		-0.00 [-0.04,0.04]		-0.00 [-0.04,0.04]		-0.00 [-0.04,0.04]
**Maternal Factors**								
Maternal BMI		-0.00 [-0.00,0.00]		-0.00 [-0.00,0.00]		-0.00 [-0.00,0.00]		-0.00 [-0.00,0.00]
Maternal education (ref: GCSE/ A-levels)								
Degree		0.00 [-0.03,0.03]		0.00 [-0.03,0.03]		-0.00 [-0.03,0.03]		0.00 [-0.03,0.03]
Higher degree		-0.00 [-0.03,0.03]		-0.00 [-0.03,0.03]		-0.00 [-0.04,0.03]		-0.00 [-0.04,0.03]
Maternal working hours (ref: not employed)								
<20 hours		0.01 [-0.03,0.05]		0.00 [-0.04,0.04]		0.00 [-0.04,0.04]		0.00 [-0.04,0.04]
21-35 hours		0.01 [-0.02,0.05]		0.01 [-0.03,0.04]		0.01 [-0.03,0.04]		0.01 [-0.03,0.04]
> 35 hours		-0.00 [-0.04,0.03]		-0.01 [-0.05,0.02]		-0.01 [-0.05,0.02]		-0.01 [-0.05,0.02]

95% confidence intervals in brackets; * $p < 0.05$, ** $p < 0.01$, *** $p < 0.001$

For peer review only - http://bmjopen.bmj.com/site/about/guidelines.xhtml
For peer review only

Supplementary Table S3: Associations between childcare attendance and environment and sum of skinfolds thickness (n=144)

	Exposure Measures β [95% CI]							
	Weekly hours in care		EPAO PA score		EPAO Nutrition score		EPAO total score	
	Unadjusted	Adjusted	Unadjusted	Adjusted	Unadjusted	Adjusted	Unadjusted	Adjusted
Constant	15.12*** [9.80,20.44]	11.66*** [6.03,17.30]	15.71*** [8.20,23.23]	10.17* [2.07,18.27]	16.38*** [9.12,23.64]	14.30*** [6.81,21.78]	15.96*** [6.75,25.17]	12.09* [2.43,21.76]
**Child factors**								
Sex	1.18* [0.18,2.19]	1.41** [0.47,2.35]	1.25* [0.23,2.27]	1.53** [0.57,2.49]	1.24* [0.22,2.26]	1.52** [0.57,2.48]	1.25* [0.23,2.27]	1.53** [0.57,2.49]
Age in months	-0.02 [-0.13,0.09]	-0.05 [-0.16,0.05]	-0.03 [-0.14,0.08]	-0.06 [-0.17,0.05]	-0.03 [-0.14,0.08]	-0.06 [-0.17,0.05]	-0.03 [-0.14,0.08]	-0.06 [-0.17,0.05]
Ethnicity (ref: white)								
White European		-0.18 [-1.94,1.58]		-0.35 [-2.17,1.48]		-0.20 [-2.00,1.61]		-0.25 [-2.07,1.58]
Other/ mixed		-0.04 [-1.51,1.42]		-0.01 [-1.51,1.50]		0.08 [-1.40,1.55]		0.10 [-1.39,1.59]
**Maternal Factors**								
Maternal BMI		0.27*** [0.15,0.40]		0.27*** [0.15,0.40]		0.27*** [0.15,0.40]		0.27*** [0.14,0.40]
Maternal education (ref: GCSE/ A-levels)								
Degree		-1.61** [-2.81,-0.41]		-1.46* [-2.67,-0.24]		-1.49* [-2.70,-0.28]		-1.49* [-2.71,-0.28]
Higher degree		-0.27 [-1.54,1.00]		0.13 [-1.13,1.40]		0.16 [-1.11,1.44]		0.09 [-1.18,1.35]
Maternal working hours (ref: not employed)								
<20 hours		-1.03 [-2.44,0.37]		-0.93 [-2.36,0.50]		-0.93 [-2.36,0.50]		-0.90 [-2.33,0.53]
21-35 hours		-0.30 [-1.64,1.04]		-0.01 [-1.34,1.31]		0.07 [-1.24,1.39]		0.04 [-1.29,1.37]
>35 hours		-0.36 [-1.82,1.09]		-0.05 [-1.42,1.32]		-0.04 [-1.41,1.33]		-0.05 [-1.42,1.33]

95% confidence intervals in brackets; * $p < 0.05$, ** $p < 0.01$, *** $p < 0.001$

For peer review only

For peer review only - http://bmjopen.bmj.com/site/about/guidelines.xhtml

STROBE 2007 (v4) Statement—Checklist of items that should be included in reports of *cross-sectional studies*

Section/Topic	Item #	Recommendation	Reported on page #
**Title and abstract**	1	(*a*) Indicate the study's design with a commonly used term in the title or the abstract	2
		(*b*) Provide in the abstract an informative and balanced summary of what was done and what was found	2
**Introduction**			
Background/rationale	2	Explain the scientific background and rationale for the investigation being reported	3-5
Objectives	3	State specific objectives, including any prespecified hypotheses	5
**Methods**			
Study design	4	Present key elements of study design early in the paper	6
Setting	5	Describe the setting, locations, and relevant dates, including periods of recruitment, exposure, follow-up, and data collection	6-7
Participants	6	(*a*) Give the eligibility criteria, and the sources and methods of selection of participants	6
Variables	7	Clearly define all outcomes, exposures, predictors, potential confounders, and effect modifiers. Give diagnostic criteria, if applicable	6-8
Data sources/ measurement	8*	For each variable of interest, give sources of data and details of methods of assessment (measurement). Describe comparability of assessment methods if there is more than one group	6-8
Bias	9	Describe any efforts to address potential sources of bias	
Study size	10	Explain how the study size was arrived at	6/ 10
Quantitative variables	11	Explain how quantitative variables were handled in the analyses. If applicable, describe which groupings were chosen and why	6--9
Statistical methods	12	(*a*) Describe all statistical methods, including those used to control for confounding	9
		(*b*) Describe any methods used to examine subgroups and interactions	9-10
		(*c*) Explain how missing data were addressed	10
		(*d*) If applicable, describe analytical methods taking account of sampling strategy	9/10
		(*e*) Describe any sensitivity analyses	9/10
**Results**			

For peer review only

Participants	13*	(a) Report numbers of individuals at each stage of study—eg numbers potentially eligible, examined for eligibility, confirmed eligible, included in the study, completing follow-up, and analysed	10
		(b) Give reasons for non-participation at each stage	10
		(c) Consider use of a flow diagram	-
Descriptive data	14*	(a) Give characteristics of study participants (eg demographic, clinical, social) and information on exposures and potential confounders	12
		10	10
Outcome data	15*	Report numbers of outcome events or summary measures	10/11
Main results	16	(*a*) Give unadjusted estimates and, if applicable, confounder-adjusted estimates and their precision (eg, 95% confidence interval). Make clear which confounders were adjusted for and why they were included	14/15
		(*b*) Report category boundaries when continuous variables were categorized	-
		(*c*) If relevant, consider translating estimates of relative risk into absolute risk for a meaningful time period	-
Other analyses	17	Report other analyses done—eg analyses of subgroups and interactions, and sensitivity analyses	14
**Discussion**			
Key results	18	Summarise key results with reference to study objectives	16
Limitations	19	Discuss limitations of the study, taking into account sources of potential bias or imprecision. Discuss both direction and magnitude of any potential bias	20/21
Interpretation	20	Give a cautious overall interpretation of results considering objectives, limitations, multiplicity of analyses, results from similar studies, and other relevant evidence	22
Generalisability	21	Discuss the generalisability (external validity) of the study results	21
**Other information**			
Funding	22	Give the source of funding and the role of the funders for the present study and, if applicable, for the original study on which the present article is based	23

*Give information separately for cases and controls in case-control studies and, if applicable, for exposed and unexposed groups in cohort and cross-sectional studies.

Note: An Explanation and Elaboration article discusses each checklist item and gives methodological background and published examples of transparent reporting. The STROBE checklist is best used in conjunction with this article (freely available on the Web sites of PLoS Medicine at http://www.plosmedicine.org/, Annals of Internal Medicine at http://www.annals.org/, and Epidemiology at http://www.epidem.com/). Information on the STROBE Initiative is available at www.strobe-statement.org.

For peer review only

BMJ Open

How does the UK childcare energy-balance environment influence 3-to-4-year-olds' anthropometry? A cross-sectional exploration

Journal:	*BMJ Open*
Manuscript ID	bmjopen-2018-021520.R2
Article Type:	Research
Date Submitted by the Author:	04-May-2018
Complete List of Authors:	Hesketh, Kathryn R.; UCL Institute of Child Health, Population, Policy and Practice; University of Cambridge, CEDAR and MRC Epidemiology Unit<br>Benjamin Neelon, Sara E.; Johns Hopkins University, Department of Health, Behavior and Society, Bloomberg School of Public Health<br>van Sluijs, Esther; University of Cambridge, CEDAR and MRC Epidemiology Unit
**Primary Subject Heading**:	Epidemiology
Secondary Subject Heading:	Public health, Sports and exercise medicine
Keywords:	Childcare environment, anthropometry, Preschool, physical activity, Nutrition < TROPICAL MEDICINE

SCHOLARONE™ Manuscripts

For peer review only

How does the UK childcare energy-balance environment influence 3-to-4-year-olds' anthropometry? A cross-sectional exploration

Authors

Kathryn R. Hesketh[1,2*]

Sara E. Benjamin-Neelon[1,2]

Esther M.F. van Sluijs[1]

[1] Centre for Diet and Activity Research and MRC Epidemiology Unit, University of Cambridge, Cambridge, UK

[2] Department of Health, Behavior and Society, Johns Hopkins Bloomberg School of Public Health, Baltimore, MD, USA

[*] Corresponding author:

Kathryn.hesketh@ucl.ac.uk

krh40@cam.ac.uk

Address: Centre for Diet and Activity Research and MRC Epidemiology Unit, University of Cambridge School of Clinical Medicine, Cambridge, UK

Telephone: 01223 769169

Word count: 5133

Figures: 0

Tables: 3

Key words: Childcare environment; physical activity; nutrition; Preschool; anthropometry

For peer review only - http://bmjopen.bmj.com/site/about/guidelines.xhtml

ABSTRACT

Objectives To assess the association between time spent in care, the childcare energy-balance environment, and preschool-aged children's Body Mass Index z-score (z-BMI), waist-to-height ratio (WHR), and sum of skinfolds thickness (SST).

Design Cross-sectional study.

Setting and participants 3-to-4-year-old children were recruited from 30 childcare centres in Cambridgeshire (UK) in 2013.

Main outcome measures Objectively measured height and weight was used to calculate BMI z-score; waist circumference and height were used to generate WHR; subscapular and tricep skinfolds were used to calculate SST. Associations between childcare attendance, the nutrition, physical activity, and overall childcare environment, and three anthropometric outcomes were explored using two-level hierarchical regression models, adjusting for demographic and family-based confounders.

Results Valid data were available for 196 children (49% female). Time spent in care, the nutrition, physical activity, and overall childcare environment were not associated with children's z-BMI score, WHR, and SST.

Conclusions Childcare environment and level of attendance were not associated with UK preschool-aged children's anthropometry. The childcare environment has been central to intervention efforts to prevent/ reduce early childhood obesity, yet other factors, including child-, family-, wider environmental, and policy-level factors warrant substantial attention when considering obesity prevention strategies for young children.

For peer review only - http://bmjopen.bmj.com/site/about/guidelines.xhtml

STRENGTHS AND LIMITATIONS OF THIS STUDY

- We used objective measures of UK preschool-aged children's anthropometric indices to provide novel information about how they are associated with childcare attendance and environment.
- This was a relatively small UK childcare-based sample, but our outcomes and exposures were normally distributed, providing sufficient heterogeneity to explore our research questions.
- We used hierarchical regression analysis to take account of study design and potential clustering at the centre level.
- We also adjusted for a number of family-level factors known to be related to children's anthropometry.
- We did not have a measure of each child's birth weight to account for children's individual growth trajectories, nor previous childcare attendance to assess prospective associations.

INTRODUCTION

In 2010, 43 million preschool-aged children worldwide were estimated to be overweight or obese, with a further 92 million at risk of overweight/obesity.[1] Although levels appear to be stabilizing,[2] obesity in childhood is associated with a range of unfavourable outcomes including type 2 diabetes, hyperlipidaemia, and psychosocial problems.[3]

Obesity is often described as an imbalance between energy intake (food consumption) and energy expenditure (physical activity) resulting in excess weight gain over time.[4] As early childhood is a period of rapid growth and development, it represents a key time for

For peer review only - http://bmjopen.bmj.com/site/about/guidelines.xhtml
For peer review only

establishing healthy energy balance related behaviours (EBRBs), which include physical activity, sedentary and dietary behaviours. While parents provide the majority of care for children before they enter school, children now spend increasing amounts of time in non-parental care prior to starting formal education.[5]

In 2014, the average enrolment rate of 3-to-5-year-olds in (pre-school) educational programmes across Organisation for Economic Co-operation and Development (OECD) countries was 80%.[5] In the UK, 3-to-4-year-old children have been entitled to 15 hours of free childcare (38 weeks per year) since 2010,[6] regardless of parental employment, and as of 2017, 3-to-4-year-olds of working parents may be eligible for up to 30 free hours per week.[7] Consequently, in 2017, 95% of UK 3-to-4-year-olds were enrolled in formal care,[8] attending for 21.7 hours per week on average.[9] Preschool-aged children, and particularly those who spend large amounts of time in formal care each week, therefore rely on childcare centres to provide a significant proportion of their food and physical activity opportunities. Elements such as staff education and training, staff behaviour on the playground, lower playground density (less children per square meter), the presence of vegetation and portable toys, and open play areas appear to be positively related to children's physical activity.[10] Likewise, childcare centres and specifically, individual childcare provider practices, appear to be associated with positive dietary behaviours in young children.[11]

Given the influence of the childcare environment on children's EBRBs, and the importance of EBRBs for children maintaining a healthy weight, it follows that time spent in childcare is likely to influence children's weight status. Indeed a number of studies assessing children during infancy and preschool suggest that children attending formal care (vs. those not in formal care) are more likely to be overweight/ obese,[12,13] with both nutrition and physical

For peer review only - http://bmjopen.bmj.com/site/about/guidelines.xhtml

activity environments contributing.[14] Recently, three systematic reviews[15–17] have been conducted to evaluate the link between childcare attendance and obesity-related outcomes, assessing a combined total of 31 papers from North America (n=19), Europe (n=9), and Asia (n=3). Despite the heterogeneous nature of childcare definitions, childcare attendance appears to be associated with increased risk of overweight/obesity,[15–17] with informal care (i.e. family-member or non-relative) most commonly found to be associated with increased weight.[15,17] Several studies did however report a decreased risk or no association between informal or centre-based childcare and children's overweight/obesity.[16,17] In general, higher intensity childcare, especially when children attended before one year, increased the overweight/obesity risk, with later (≥3 years old) enrolment in centre-based care associated with lower risk of overweight/obesity compared to earlier enrolment.[17] Lastly, there is some uncertainty as to whether risk differs by socio-demographic factors: children from lower socio-economic backgrounds who spend time in childcare have been shown to have both a greater[18] and lower[19] risk of developing obesity.

Due to the high numbers of children attending formal childcare settings, and therefore the ability to reach large numbers of children in these environments, intervention studies are often conducted in these settings to prevent, halt or reverse obesity during the preschool years. A review by Zhou and colleagues[20] identified 15 such studies: six (of 13) interventions with a dietary component, and eight (of 12) interventions with physical activity component reported improvements in the target EBRB,[20] with seven (47%) reporting subsequent improvements in children's adiposity. Few studies (4/15) assessed long-term efficacy or sustainability (5/15) and the authors also noted heterogeneity across study designs and the interventions delivered, which suggests effects may be limited to specific population subgroups.

For peer review only - http://bmjopen.bmj.com/site/about/guidelines.xhtml

Such findings perhaps reflect differences in policies and practices around nutrition and physical activity across childcare centres (e.g. by level of deprivation[21]) and between countries, which likely influence the extent to which the childcare environment is associated with children's weight indicators. Moreover, uptake of childcare varies between countries,[5] and so too does the amount of time children spend in any form of care,[18] which may also influence the strength of the association between the childcare environment and obesity levels. Finally, it is possible that despite the influence of the childcare environment, parental or family-level factors, which are often targeted simultaneously, may exert a strong or stronger influence on children's weight status.[22]

In England, over 1 in 5 children are overweight or obese by the age of five.[23] Despite high levels of childcare attendance in the United Kingdom (UK), there has been very little research to assess associations between the childcare environment and children's health outcomes. With the publication of the UK Government's Obesity Plan for Action in 2017, strategies to encourage positive health behaviours and weight in preschool-aged children are increasingly centred on the childcare environment.[24] It is therefore timely to determine associations between the UK childcare environment and children's anthropometric indices. In this exploratory study, we therefore sought to assess how the amount of time spent in childcare, and how the nutrition, physical activity and overall childcare environment are associated with anthropometric indicators (z-BMI score; waist-to-height ratio (WHR); sum of skinfold thickness (SST)) in a sample of UK 3-to-4-year-old children, adjusting for a range of family-level explanatory variables. We hypothesised that children attending childcare centres with more supportive physical activity and nutrition environments would have favourable

For peer review only - http://bmjopen.bmj.com/site/about/guidelines.xhtml

(i.e. lower) anthropometric indices compared to those attending centres with less supportive environments.

MATERIALS AND METHODS

Study design and recruitment

Data were from the "Studying Physical Activity in preschool-aged Children and their Environment (SPACE) Study", a cross-sectional childcare-based observational study[25]. The STrengthening the Reporting of OBservational studies in Epidemiology (STROBE) protocol was followed in the conduct and dissemination of this observational study. Participants were not involved in the development of the study design, research questions or outcome measures, but results were disseminated to all participants and participating childcare centres. Recruitment and data collection took place in January-July 2013; detailed information is available elsewhere.[25] Briefly, a list of preschool (state-run education) and nursery (privately-run) 'childcare centres' in Cambridge were obtained from the Ofsted government website[26] and stratified by type (preschool/ nursery) and tertile of index of multiple deprivation (IMD; an area-level measure of deprivation[27]). Preschools and nurseries, but not home/ family-based children centres, were purposively recruited because funding, the built environment and care provided tend to differ by type.[25] Within these six strata, 88 childcare centres were approached at random and invited to participate in writing; 30 (34%) centre managers consented to participate (n=15 preschool and n=15 nursery).

The parents of all children aged 3-4 years (n=602) attending consenting centres were sent a study invitation pack, and requested to return written consent to the childcare centre. Eligible children were: aged 3 or 4 years; free from physical disability; attended the centre for at least nine hours per week (to ensure children spent >50 % of their government-paid allocation at

For peer review only - http://bmjopen.bmj.com/site/about/guidelines.xhtml

that particular centre); and registered to attend the childcare centre on the designated measurement day. At least five children per centre with valid written consent (by a parent/legal guardian) were required to ensure sufficient analytical power. Children provided verbal assent prior to measurement (n=247, 41%). The University of Cambridge Psychology Ethics Committee provided ethical approval for the study (Pre.2012.68).

Patient and Public Involvement

Participants were not involved in the development of the study design, research questions or outcome measures, but results were disseminated to all participants and participating childcare centres.

Data collection

Child Anthropometry and Demographic data

At each centre visit, one of three trained researchers recorded each child's sex; measured height to the nearest 0.1cm using a Leicester stadiometer; and weight to the nearest 0.1kg using Seca digital scales in light indoor clothes with shoes and socks removed. Measures of weight and height were conducted once as these are highly reproducible with limited variability.[28] Abdominal waist circumference was measured to the nearest 0.5cm at the midpoint between the lower costal margin and the level of the anterior superior iliac crests. Up to three measures were taken (if there was a discrepancy of 1cm in the first two measures) using a Seca non-stretch tape measure next to the child's skin. Subscapular and tricep skinfolds were measured on the child's right side using a Holtain caliper (Holtain Ltd, United Kingdom) according to standard protocol.[29] If the first two measurements at either site were >0.2mm apart, a third measurement was taken and an average calculated. Compared to a 'gold standard' trainer, researcher mean differences in measurement was 0.1cm and 0.25-

For peer review only - http://bmjopen.bmj.com/site/about/guidelines.xhtml

0.3mm for waist and skinfolds respectively. Equipment was calibrated prior to commencing data collection, at the mid-way point and on completion of the study.

Following anthropometric measurement, each child was allocated a study pack containing a parental questionnaire, which care providers disseminated to parents. The parental questionnaire, based on a previously validated questionnaire,[30] assessed demographic factors relating to the study participant; their general health and common health behaviours; childcare attendance; other children in the home; family socio-demographics; parental occupational and leisure physical activity; parental height and weight; and parental beliefs, barriers and attitudes towards physical activity and nutrition. Parents were asked to return the questionnaire to their child's childcare centre one week later.

Assessment of Childcare Environment

A trained researcher assessed the physical activity and nutrition environment of each centre using the Environment and Policy Assessment and Observation (EPAO) instrument. Observation began when the first child arrived in the morning and continued until the end of the day, when the last child left,[31] as the EPAO protocol requires a minimum of one full day to be spent observing all activities in the designated childcare classroom. Scoring is composed of two eight-item subscales for physical activity and nutrition. The physical activity subscales comprise: Active Opportunities, Sedentary Opportunities, Sedentary Environment, Portable Play Environment, Fixed Play Environment, Staff Physical Activity Behaviours, Physical Activity Training and Education, and Physical Activity Policy. Nutrition subscales comprise: Servings of fruits and vegetables; Whole grains; High-sugar/high-fat foods; Beverages; Staff nutrition behaviours; Nutrition environment; Nutrition training and education; and Nutrition policy.

For peer review only - http://bmjopen.bmj.com/site/about/guidelines.xhtml

As this tool was developed in the USA, a number of small amendments to the protocol and data collection template were made to ensure the tool was suitable in the UK context. This was done in consultation with the original development team to ensure the measure appropriately captured the physical activity and nutrition environments in UK childcare centres. These amendments did not change how the EPAO subscales for physical activity were scored; where preschools did not serve food, we used averages of available variables to derive the nutrition subscale scores.

Variable derivation

Outcome measures

Objectively measured height and weight were used to calculate children's BMI (weight[Kg]/ height^2[m]). This, combined with child's sex and age in months at measurement (calculated from parental reported date of child's birth), was used to derive a continuous BMI z-score, based on the British 1990 (UK90) growth reference charts.[32] International Obesity Task Force classifications were used to categorise children as normal weight, overweight or obese.[33] Waist-to-height ratio (WHR) (waist [cm]/ height [cm]) was derived to assess central obesity, and the sum of subscapular and tricep skinfold thicknesses (SST) was derived as an indicator of subcutaneous fat.[34]

Exposure variables

Parents were asked to provide information about their child's usual weekly childcare attendance using a specifically designed question:[25] "In a usual week, when does your child attend childcare? *Please only include care for your child taking part in SPACE and include regular formal and informal care (e.g. grandparents, friends etc.).*" Parents

For peer review only - http://bmjopen.bmj.com/site/about/guidelines.xhtml

responded using free text, which we subsequently coded to derive the total number of reported hours children attended *formal* childcare during a usual week for analyses. Indicators of the overall childcare, nutrition, and physical activity environments using EPAO (sub-scale) scores were also generated. According to standard EPAO scoring procedure, responses to questions across eight physical activity, and up to eight nutrition sub-domains were summed to a possible maximum of 20 points per domain (where each question was worth 0-2 points). The 'Physical Activity' domain score was derived using an average of the eight subscale domain scores for all centres. The 'Nutrition' domain score was calculated using an average of six **or** eight subdomain scores, depending on meals served. Many UK childcare centres do not serve all meals (i.e. some only serve snacks/ require children to bring packed lunch, and do not provide lunch and 'tea' (at ~4pm)). Two nutrition subscale scores (i.e. Whole grains; High-sugar/high-fat foods) could therefore not be calculated for 21 centres: for these an average of the relevant six subdomain scores was calculated. An overall 'EPAO score' was derived by averaging the eight physical activity, and six or eight nutrition subdomain scores.

Additional confounding variables

A range of potential explanatory variables relating to the child's family environment were derived using data collected from a parental questionnaire, including child ethnicity (White British; White European; Other); maternal educational attainment (General Certificate of Secondary Education (GCSEs); A Levels; National Vocational Qualification (NVQ)/ Diploma; University degree; Higher degree); maternal self-report BMI; and hours per week mothers worked (Not employed; ≤20 hours; 21-35 hours; >35 hours). Where available, the latter three variables were also derived for fathers (with hours per week fathers worked categorised based on distribution as: <40 hours; 40-42 hours; >42 hours).

For peer review only - http://bmjopen.bmj.com/site/about/guidelines.xhtml

Statistical Analyses

All analyses were conducted using STATA 14/SE. We calculated descriptive statistics for included participants, and compared them to those excluded from analyses (i.e. those without valid anthropometric data) using independent t-tests and Pearson's chi^2.

For each of our three continuous outcome variables (z-BMI score, WHR, SST), a series of two-level mixed-effects linear regression analyses (level 1: child; level 2: childcare centre) were conducted to explore how weekly childcare attendance and practices relating to energy-balance behaviours in the childcare environment were related to children's anthropometric indices. Given children's age in months and sex are already taken into account when z-BMI score is derived, all WHR and SST analyses were adjusted for these variables. As children in the SPACE study were recruited or 'clustered' at the centre level, multi-level regression analyses were used to allow for both within- and between-centre variation in anthropometric outcomes.[25,35] First, univariate analyses were conducted to assess how a) weekly childcare attendance (in hours), and b) the childcare environment relating to nutrition and physical activity, were associated with each of the three anthropometric indices. Multivariable analyses were then run, adjusting for family-level confounding variables. One centre contributed two classes, which were treated as separate centres in analyses: although the classes shared policy documents, each ran as a completely independent entity, with different staff, rooms, children and outside spaces, and did not share catering facilities (hot meals were not provided).

Significant within-centre differences across the three outcome measures were identified. For example, 96% of the variation in z-BMI score was explained by within-centre (i.e. child-level) differences and only 4% of variation was explained by between-centre (i.e. childcare-

For peer review only - http://bmjopen.bmj.com/site/about/guidelines.xhtml

level) differences [within-centre variation WHR: 73%; STT: 85%]. To further explore these differences, *post hoc* analyses were conducted to determine whether child-level socio-economic status (measured using maternal educational attainment) moderated the association between time spent in the childcare environment and each outcome. Based on previous evidence, we hypothesised that the relationship between a more favourable childcare environment and normal weight would be stronger (i.e. the gradient would be steeper) for children in lower income families. This was grounded on the assumption that favourable childcare environments would buffer the association between potentially negative home environments and overweight/ obesity in children from poorer socio-economic backgrounds, but that the childcare environment may be less important for children from higher income homes. Also, as fewer children provided SST data than z-BMI score and WHR, sensitivity analyses were conducted to limit our analyses to children providing complete case valid data for all three outcomes (n=151).

RESULTS

Participant Characteristics

Of the 247 children who assented to have anthropometric measurements taken, valid anthropometric, observational, and questionnaire data were available for 196 children from 30 centres (Table 1). Childcare centres have been described previously;[36] those centres who participated did not differ in terms of area-level socio-economic characteristics from those who declined to participate.[25] Children who provided complete case data (n=196) did not differ from those excluded by child's sex, age, weight status or ethnicity but were more likely to have higher educated mothers (higher degree: 38.3% vs. 22.2%, p=0.02).

For peer review only - http://bmjopen.bmj.com/site/about/guidelines.xhtml

Table 1 Descriptive characteristics of children included in analyses (SPACE study, 2013, N=196)

	Boys	Girls
**Child Characteristics**		
N (%)	100 (51)	96 (49)
Age (in months)	47.4 (5.2)	47.8 (4.9)
Ethnicity (N(%))		
White British	74 (74.0)	74 (77.1)
White other	9 (9.1)	9 (9.4)
Other/ mixed ethnicity	17 (17.0)	13 (13.5)
BMI z-score	0.52 (0.93)	0.36 (1.06)
Weight category[a] (N(%))		
Normal	82 (82.0)	79 (82.3)
Overweight/obese	18 (18.0)	17 (17.7)
Waist/ Height ratio	0.49 (0.1)	0.49 (0.1)
Centrally obese (N(%))	35 (46.7)	28 (40.6)
Sum of skinfolds[b] (cm)	14.5 (3.1)	16.0 (3.6)
Average weekly hours in childcare	23.3 (12.2)	21.8 (12.1)
**Maternal Characteristics**		
Age (in years)	37.6 (5.0)	37.4 (5.5)
BMI (in kg/m^2)	23.9 (4.2)	24.2 (4.7)
Education (N(%))		
GCSE/A-levels	25 (25.0)	34 (35.4)
Degree	29 (29.0)	33 (34.4)
Higher degree	46 (46.0)	29 (30.2)
Hours worked per week[c] (N(%))		
Not employed	26 (27.4)	24 (25.8)
<20 hours	19 (20.0)	14 (15.1)
21-35 hours	28 (29.5)	31 (33.3)
>35 hours	22 (23.2)	24 (25.8)
**Paternal Characteristics[e]**		
Age (in years)	39.2 (5.6)	39.4 (8.0)
BMI (in kg/m^2)	25.2 (3.5)	25.9 (3.2)
Paternal education (N(%))		
GCSE/A-levels	23 (23)	20 (24.1)
Degree	24 (24)	27 (32.5)
Higher degree	43 (43)	36 (43.4)
Hours worked per week[c] (N(%))		
<40 hours	26 (29.8)	30 (35.3)
40-42 hours	30 (35.6)	30 (36.6)
>42 hours	30 (34.5)	22 (26.8)

All values mean(SD) unless stated otherwise; BMI: Body mass index; a: Weight category derived using the International Task Force on Obesity cut points; GCSE: General Certificate of Secondary Education; A-levels: Advanced Levels; b: n=80 boys and n=71 girls; c: categorized based on distribution; c: maternal employment: n=95 for boys, n=93 for girls; d: paternal variables available for n=169-173 children depending on variable

For peer review only - http://bmjopen.bmj.com/site/about/guidelines.xhtml

Childcare Environment

Domain subscale and total scores for the EPAO are shown in Table 2. Across childcare centres, the mean total EPAO score (including eight physical activity and up to eight nutrition domains) was 11.2 (SD: 1.0, range: 8.5 - 13.5), with higher scores signifying more supportive environments. The average physical activity subscale score was 10.8 (1.5, 7.4-13.8) and average nutrition subscale score was 11.7 (1.6, 8.8-14.3). Overall, nutrition scores indicated good provision of fruit and vegetables, limited servings of high-fat/high-sugar foods, but poor provision of wholegrains. For physical activity, centres generally scored well for active opportunities and on staff physical activity behaviours; staff training and education in physical activity, and physical activity policies, were largely lacking.

For peer review only

Table 2 EPAO average domain subscale and total scores in the SPACE study

Nutrition sub-domains[a]	Mean (SD)	Range	Physical Activity sub-domains[b]	Mean (SD)	Range
Servings: fruits and vegetables	13.4 (4.1)	6.7-20.0	Active Opportunities	15.3 (3.8)	6.7 – 20.0
Servings: Whole grains[c]	3.3 (3.3)	0-10.0	Sedentary Opportunities	11.7 (4.5)	6.7 – 20.0
Servings: High-sugar/high-fat foods[d,e]	14.5 (0.9)	14.0 –16.0	Sedentary Environment	12.7 (4.1)	6.7 – 20.0
Servings: Beverages	9.7 (2.4)	5.0 - 16.3	Portable Play Environment	10.7 (3.1)	5.7 - 17.1
Staff nutrition behaviours	10.1 (2.8)	3.3 -15.0	Fixed Play Environment	11.8 (2.8)	7.5 - 16.3
Nutrition environment	15.4 (3.0)	6.7 – 20.0	Staff PA Behaviours	14.3 (2.5)	8.0 – 20.0
Nutrition training and education	10.6 (3.1)	4.0 – 18.0	PA Training and Education	4.8 (4.0)	0 - 15.0
Nutrition policy	12.6 (6.2)	6.7 – 20.0	PA Policy	7.0 (6.8)	0 - 20.0
**Total Domain Score[b]**					
Average Nutrition domain score[f]	11.7 (1.6)	8.8 - 14.3	Average Physical Activity domain score[g]	10.8 (1.5)	7.4 - 13.8
**Total EPAO Score[b]**					
Average total EPAO score[h]	11.2 (1.0)	8.5 - 13.5			

EPAO: Environment and Policy Assessment and Observation tool; a: n=30 unless stated; b: n=30; c: centres n=9; d: centres n=8; e: reverse coded such that higher score means favourable or lower provision of high-sugar, high-fat foods; f: average of up to 8 subdomain scores; g: includes 8 subdomain scores; h: average of up to 8 nutrition and 8 physical activity domain scores

For peer review only - http://bmjopen.bmj.com/site/about/guidelines.xhtml

Association between childcare environment and children's anthropometric indices

In unadjusted analyses, there was no significant association between the number of hours a child spent in care or energy-balance childcare practices and preschool-aged children's anthropometric indices (Table 3). These findings remained unchanged after adjusting for family-level variables; as expected, several family-level variables were independently associated with the outcomes of interest in adjusted models (see supplementary Table S1-S3).

We found no significant interactions in *post hoc* analyses, designed to determine whether child-level socio-economic status (measured using maternal educational attainment) moderated the association between time spent in the childcare environment and our three outcome measures (data not shown). In sensitivity analyses, including only children who provided all three outcome measures (n=151) did not significantly influence our findings (data not shown).

For peer review only

For peer review only - http://bmjopen.bmj.com/site/about/guidelines.xhtml

Table 3 Associations between childcare environment and preschool-aged children's anthropometric indices in the SPACE study

	Exposure measures β [95% CI]							
	Weekly hours in care		EPAO PA score		EPAO Nutrition score		EPAO total score	
**Continuous Outcomes**	Unadjusted	Adjusted[a]	Unadjusted	Adjusted[a]	Unadjusted	Adjusted[a]	Unadjusted	Adjusted[a]
BMI z-score[b]	-0.00	-0.01	-0.02	-0.01	0.01	-0.01	-0.00	-0.00
	[-0.02,0.01]	[-0.02,0.00]	[-0.12,0.09]	[-0.12,0.11]	[-0.09,0.10]	[-0.11,0.09]	[-0.01,0.01]	[-0.01,0.01]
Waist to height ratio[b,c]	-0.00	-0.00	0.01	0.00	-0.00	-0.00	0.00	0.00
	[-0.00,0.00]	[-0.00,0.00]	[-0.01,0.02]	[-0.01,0.02]	[-0.01,0.01]	[-0.01,0.01]	[-0.00,0.00]	[-0.00,0.00]
Sum of skinfold[c,d]	0.02	0.03	0.03	0.18	-0.03	-0.18	0.00	0.00
	[-0.02,0.07]	[-0.01,0.08]	[-0.44,0.50]	[-0.30,0.67]	[-0.45,0.40]	[-0.61,0.26]	[-0.05,0.05]	[-0.05,0.05]

EPAO: Environment and Policy Assessment and Observation tool; a: model adjusted for child ethnicity, maternal BMI, maternal educational attainment and maternal working hours; b: n=196; c: model also includes child sex and age in months; d: n=144

For peer review only - http://bmjopen.bmj.com/site/about/guidelines.xhtml

For peer review only

For peer review only

For peer review only - http://bmjopen.bmj.com/site/about/guidelines.xhtml

DISCUSSION

We found that childcare attendance and energy-balance practices in the childcare environment do not appear to be associated with UK preschool-aged children's anthropometric indices. However, as shown previously, family-level factors were independently associated with children's z-BMI score. Children spend increasing amounts of time in formal childcare in the UK and the childcare environment is frequently the focus of intervention efforts to prevent or reduce early childhood obesity worldwide.[24] Childcare centres in the UK adhere to a statutory Early Years Foundation Stage (EYFS) framework, operate 'free-flow' policies where children generally choose their own activities with few provider-led activities, and by law must ensure all food and drink provided is properly prepared, wholesome and nutritious.[37] Therefore, this relatively standardised level of care may mean that UK childcare environments exert a smaller influence on children's EBRBs and health. Together, this suggests that child-, family-, environmental-, and policy-level factors warrant significant further attention in obesity prevention strategies for young children.

How this work compares to previous studies

Cross-sectionally, we did not identify any associations between time spent in care and children's anthropometry. This is in accordance with previous studies conducted in low-income Latino 4-year-olds[38] and 2-5-year olds in Australia[39]. Interestingly, the latter study identified maternal education as the sole significant predictor of child's weight status.[39] Although consideration of family level factors do not impact the conclusions drawn here (i.e. no association was found before or after adjustment), several were independently associated with BMI z-score, significantly attenuating the (non-significant) relationship between exposure and outcome. This confirms previous findings that family

For peer review only - http://bmjopen.bmj.com/site/about/guidelines.xhtml

level factors may influential to children's weight status, regardless of childcare attendance.[22]

Within the SPACE study, there was relatively wide heterogeneity in design and layout of childcare centres. Yet EPAO scores in this study showed comparatively little variation across childcare centres and were similar to those seen previously in US,[40] Dutch,[36] and Canadian[41] studies which suggests there were no obvious differences between these UK and other childcare environments. However, across and between countries differences may exist in how 'healthy' childcare environments are, and there may also be variation across physical activity and nutrition environments within the same childcare environment (i.e. good nutrition, poor physical activity and vice versa). Taken together, the definition of a 'healthy' environment is likely to be relatively heterogeneous, potentially preventing identification of consistent associations.

It could be hypothesised that childcare in earlier childhood (i.e. infancy), rather than during the preschool-period (as assessed here), is more important for the development of children's adiposity. Indeed, work conducted in prospective cohorts suggests that early childcare attendance may be associated with children's weight status: Black and colleagues noted that early informal care (before 3 years) was associated with increased risk of overweight/obesity, and that higher childcare attendance, especially starting before 1 year, also increased overweight/obesity risk.[42] This was confirmed in studies in younger children (aged 1-2 years) from Ireland and The Netherlands. The latter suggest that attending childcare part- or full-time was associated with increases in anthropometric indicators and odds of being overweight compared to those not in childcare prospectively.[43,44] Moreover, cumulative exposure to centre-based care up to the age of 4 appears to be associated with higher odds of overweight/obesity in later childhood, i.e. at 4-10 years of age,[12] in Canadian children. As

For peer review only - http://bmjopen.bmj.com/site/about/guidelines.xhtml
For peer review only

such, these longitudinal associations appear to be dependent on the timing and level of exposure to childcare.[15,42] Greater childcare attendance in very early childhood appears to be associated with childhood obesity, but later childcare attendance does not show such associations.[42] As no data on previous childcare attendance patterns were collected in this study, we were unable to test this hypothesis here.

It is conceivable that bias or residual confounding, in addition to our sample size, limited our ability to find associations; it is also possible that a lack of power (i.e. low β) resulted in a type 2 error, or us failing to find an association where one existed. This said, we were able to detect relatively small associations between the outcome and family level factors (e.g. maternal BMI: β co-efficient=0.05), and had suitable heterogeneity in our exposure measures to do so. Previous studies have identified associations with a range of time-based exposures assessed over longer periods of time (i.e. 10 hours/week increments;[45] each additional 30 days exposure to care[13] during the first year of life) or simply by the type of care children were exposed to (i.e. childcare vs. informal vs. parental care[12,18]).[15] This variation in exposure and outcome measures is therefore also likely to account for differing findings between childcare-related factors and children's anthropometric indices.

This is to our knowledge one of the few studies to go beyond exploration of the association between time spent in care and anthropometry, to assess how elements within the childcare environment (i.e. nutrition and physical activity practices) are related to a children's anthropometric indices. In a previous study using this same sample of children, we found a similar lack of association between the childcare environment and preschool-aged children's physical activity[36] as identified here between the preschool environment and children' anthropometry. This is in contrast to studies in the US[40] and Canada[46] suggesting that

For peer review only - http://bmjopen.bmj.com/site/about/guidelines.xhtml

more favourable physical activity environments are associated with higher physical activity levels in children. It is possible that differences identified here are due in part to a fairly standardised system of care in the early years in the UK mentioned earlier. The EYFS framework [47] is a curriculum against which children's development is judged at age 2 and 5 years (after their first year in primary school). A key tenant of this is that children must "be helped to understand the importance of physical activity, and to make healthy choices in relation to food." (Pg 5;[47]) All food and drink served to children in UK early years settings must by law be properly prepared, wholesome and nutritious, with fresh drinking water available to children at all times.[37] UK childcare centres additionally operate a 'free-flow' policy were children may choose from a range of inside and outside activities for the majority of the day, regardless of weather conditions. With a standardised level of provision, the childcare environment in the UK may exert only a small influence on children's EBRBs and health outcomes. Yet with a core mandate to promote healthy EBRBs, and with high levels of children attending childcare, childcare settings in the UK appear well placed to ensure all children receive the best start in life, irrespective of socio-demographic factors.

To date, much of the research in this area has focused on the formal childcare environment. Strategies to encourage positive health behaviours and weight in preschool-aged children are therefore increasingly centred on the childcare environment, particularly in the UK.[24] However, very little research has been conducted in the UK childcare setting to determine whether such interventions are necessary or likely to succeed. Indeed, this and previous research[36] suggests that the formal childcare environment in the UK does not appear to be associated with preschool-aged children's anthropometric and physical activity outcomes. Other types of care (e.g. family child care homes/ childminder; grandparents) however also deserve attention. For example in Canada, preschool-aged children in full day kindergarten

For peer review only - http://bmjopen.bmj.com/site/about/guidelines.xhtml

(providing instructive programmes for preschoolers) accumulated significantly more MVPA than those in centres (providing developmental programmes for a range of ages) or home-base care,[46] whereas in another study, EPAO nutrition subscales in centre-based care were shown to be more supportive than those in home-based care.[41] Given UK preschool-aged children spent an average of 15 hours in informal care in 2017,[9] more research is needed into the potential influences of these informal environments. This said, areas for improvement within the formal childcare environment likely still exist, with a more targeted approach, focusing on specific areas within the childcare environment (e.g. promoting healthy dietary provision[21], improving physical activity policies and staff training), still potentially benefitting children's health behaviours.

Finally, the children's family and wider environments should not be overlooked. Although the family is suggested to be central to health promotion in young children, both more proximal family and external factors (e.g. in the community and wider environment) combine to shape a child's health behaviours.[48] Indeed, parents and childcare providers often cite each other as important custodians of preschool-aged children's EBRBs, suggesting both should work synergistically to encourage positive health and habit formation.[49]

Strengths and limitations

We used objective measures of UK preschool-aged children's anthropometric indices to provide novel information about how attendance and the childcare environment are associated with children's anthropometric indices. Although this was a relatively small UK childcare-based sample, both our outcomes and exposures were normally distributed, and provided us with sufficient heterogeneity to explore our research questions. Although derivation of one of the exposure measures (i.e. the Nutrition domain score) differed from

For peer review only - http://bmjopen.bmj.com/site/about/guidelines.xhtml

previous studies, limiting the number of sub-scale scores used (to 6 rather than taking an average of up to 8) did not influence our findings. As one trained observer conducted the EPAO ratings, it is possible that bias or inaccuracy in these ratings may have occurred; the impact of this on the EPAO predictor variables is unknown. By using hierarchical regression analysis, we were also able to take account of study design and potential clustering at the centre-level, thus increasing our power to detect (small) significant differences in our outcomes. We also adjusted for a number of family-level factors known to be related to children's anthropometry but we did not have a measure of each child's birth-weight to account for children's individual growth trajectories. As with several studies in this area,[15] this work was based on childcare-based sample. We were therefore unable to assess the influence of no or only informal care, in addition to centre-based care, on children's weight-related outcomes.

Children were drawn from childcare centres recruited from the top tertile of Index of Multiple Deprivation (IMD) scores in England.[25] As there were no differences in IMD scores of the centres that did and did not participate in the SPACE study,[36] children included here are likely representative of the wider eligible population. Children's individual socio-economic circumstances (i.e. maternal educational attainment) varied within this sample, which is important as children from both lower and higher socio-economic backgrounds may be at risk of higher BMI.[18,19,23] It is not therefore not clear how generalizable our findings are. However, we did not see evidence of a moderating effect of children's socio-economic circumstances on the exposure-outcome relationship, suggesting that the association may be consistent across socio-economic strata. Just under one-fifth (18%) of children were classified as overweight or obese, which is slightly below the national UK average for 5-year old children (22.5% in 2013/14).[23] Children were predominantly

For peer review only - http://bmjopen.bmj.com/site/about/guidelines.xhtml

White (British/ European) (85%), which is in line with the UK average (86% in 2011).[50] Work is however required to determine whether similar findings are apparent in differing minority populations across the UK.

For peer review only

For peer review only - http://bmjopen.bmj.com/site/about/guidelines.xhtml

CONCLUSIONS

We found no significant association between childcare attendance, or the nutrition and physical activity childcare environment, and children's anthropometric outcomes, suggesting the UK childcare environment had little influence on children's weight status in our study. In contrast, family-level factors were associated. Although childcare-based interventions are increasingly the focus for promoting healthy weight and EBRBs in preschool-aged children, they tend to show small effects that are not sustained over time. Looking to other areas of a child's life, specifically family-level factors and those in a child's wider environment, either as an adjunct or alternative to centre-based interventions, should become a focus. Considering how a broader range of potential influences may interact to contribute to children's health will be key in successfully promoting healthy weight in preschool-aged children.

For peer review only

ACKNOWLEDGMENTS

We thank all children and their parents who participated in the SPACE study. In addition, we thank members of the MRC Epidemiology Unit for their assistance: to members of the field team who conducted data collection, to Kate Westgate and Stefanie Hollidge from the physical activity technical team for the accelerometer data processing, and to Dr Emanuella De Lucia Rolfe and Richard Powell for processing the anthropometric data.

CONTRIBUTORSHIP

KH and EvS conceived of the SPACE study. KH collected the data, conducted the analyses and drafted the manuscript. KH, SBN and EvS interpreted the results and critically revised the manuscript. All authors approved the final manuscript and agree to be accountable for all aspects of the work.

FUNDING

This work was conducted by the Medical Research Council [Unit Programme number MC_UU_12015/7] and the Centre for Diet and Activity Research (CEDAR), a UKCRC Public Health Research Centre of Excellence. Funding from the British Heart Foundation, Cancer Research UK, Economic and Social Research Council, Medical Research Council, the National Institute for Health Research, and the Wellcome Trust, under the auspices of the UK Clinical Research Collaboration, is gratefully acknowledged (CEDAR grant numbers: ES/G007462/1; 087636/Z/08/Z; MR/K023187/1). KH is funded by the Wellcome Trust (107337/Z/15/Z). SBN is funded by NIH and the Robert Wood Johnson Foundation.

COMPETING INTERESTS

All authors have completed the ICMJE uniform disclosure form at http://www.icmje.org/coi_disclosure.pdf (available on request from the corresponding author) and declare that (1) they have support from a number of grants outlined above; (2) all authors have no relationships with any organisations that might have an interest in the submitted work in the previous 3 years; (3) their spouses, partners or children have no financial relationships that may be relevant to the submitted work and (4) have no non-financial interests that may be relevant to the submitted work.

For peer review only - http://bmjopen.bmj.com/site/about/guidelines.xhtml

ETHICS APPROVAL

Ethical approval was granted by The University of Cambridge Psychology Ethics Committee for the study (Pre.2012.68).

DATA SHARING STATEMENT

Unpublished data regarding children's health behaviours, socio-economic circumstances and parental behaviours are available for the SPACE study. The anonymised data set and statistical code used for analyses are available from the corresponding author on request. Model parameters are outlined in the 'Methods' section. Participants gave assent and parents provided informed consent for study participation, but the presented data are anonymised and risk of identification is very low.

For peer review only

REFERENCES

1 de Onis M, Blossner M, Borghi E. Global prevalence and trends of overweight and obesity among preschool children. *Am J Clin Nutr* 2010;92:1257–64.

2 Rokholm B, Baker J, Sørensen T. The levelling off of the obesity epidemic since the year 1999 - a review of evidence and perspectives. *Obes Rev* 2010;11:835–46.

3 Lakshman R, Elks CE, Ong KK. Childhood Obesity. *Circulation* 2012;126:1770–9.

4 World Health Organisation. Obesity and overweight. WHO Fact sheet N°311. 2013. http://www.who.int/mediacentre/factsheets/fs311/en/ (accessed 3 Mar 2017).

5 OECD. Equity and Quality in Education: Supporting Disadvantaged Students and Schools. OECD. 2012. http://dx.doi.org/10.1787/9789264130852-en (accessed 3 Feb 2015).

6 Office for Standards in Education (Ofsted). Early Years and Childcare. www.gov.uk. 2016. http://www.ofsted.gov.uk/early-years-and-childcare (accessed 30 Dec 2013).

7 UK Government. Help paying for childcare. 2018. https://www.gov.uk/help-with-childcare-costs/free-childcare-and-education-for-2-to-4-year-olds (accessed 2 Mar 2018).

8 UK Government. Education provision: children under 5 years of age, January 2017. Childcare and Early Years. 2017. https://www.gov.uk/government/statistics/education-provision-children-under-5-years-of-age-january-2017 (accessed 28 Feb 2018).

9 Department for Education. Childcare and early years survey of parents: 2017. https://www.gov.uk/government/uploads/system/uploads/attachment_data/file/669857/SFR73_2017_Text.pdf (accessed 9 Mar 2018).

10 Trost SG, Ward DS, Senso M. Effects of child care policy and environment on physical activity. *Med Sci Sport Exerc* 2010;42:520–5.

11 Gubbels JS, Gerards SMPL, Kremers SPJ. Use of food practices by childcare staff and

For peer review only - http://bmjopen.bmj.com/site/about/guidelines.xhtml

the association with dietary intake of children at childcare. *Nutrients* 2015;7:2161–75.

12 Geoffroy M-C, Power C, Touchette E, et al. Childcare and overweight or obesity over 10 years of follow-up. *J Pediatr* 2013;162:753–758.e1.

13 Benjamin Neelon SE, Schou Andersen C, Schmidt Morgen C, et al. Early child care and obesity at 12 months of age in the Danish National Birth Cohort. *Int J Obes* 2015;39:33–8.

14 Sisson SB, Li J, Stoner JA, et al. Obesogenic environments in tribally-affiliated childcare centers and corresponding obesity rates in preschool children. *Prev Med Reports* 2016;3:151–8.

15 Alberdi G, McNamara AE, Lindsay KL, et al. The association between childcare and risk of childhood overweight and obesity in children aged 5 years and under: a systematic review. *Eur J Pediatr* 2016;175:1277–94.

16 Costa S, Adams J, Gonzalez-nahm S, et al. Childcare in Infancy and Later Obesity : a Narrative Review of Longitudinal Studies. *Curr Pediatr Reports* 2017;5:118–31.

17 Black L, Kearney PM. The association between childcare arrangements and risk of overweight and obesity in childhood: a systematic review. *Obes Rev* 2017;18(10):1170-1190 18

18 Pearce A, Li L, Abbas J, et al. Is childcare associated with the risk of overweight and obesity in the early years? Findings from the UK Millennium Cohort Study. *Int J Obes* 2010;34:1160–8.

19 Nazarov Z, Rendall M. Differences by mother's education in the effect of childcare on child obesity. *Econ Lett* 2014;124:286–9.

20 Zhou YE, Emerson JS, Levine RS, et al. Childhood obesity prevention interventions in childcare settings: Systematic review of randomized and nonrandomized controlled trials. *Am J Heal Promot* 2014;28:92–104.

For peer review only - http://bmjopen.bmj.com/site/about/guidelines.xhtml

21 Neelon SEB, Burgoine T, Hesketh KR, et al. Nutrition practices of nurseries in England: Comparison with national guidelines. *Appetite* 2015;85:22-9.

22 Hawkins SS, Cole TJ, Law C. An ecological systems approach to examining risk factors for early childhood overweight: findings from the UK Millennium Cohort Study. *J Epidemiol Community Health* 2009;63:147–55.

23 NHS Digital. National Child Measurement Programme - England, 2016-17. https://digital.nhs.uk/catalogue/PUB30113 (accessed 4 Nov 2017).

24 Department of Health. Childhood Obesity: A Plan for Action. London, UK: 2016. https://www.gov.uk/government/uploads/system/uploads/attachment_data/file/546588/Childhood_obesity_2016__2__acc.pdf (accessed 4 Nov 2017).

25 Hesketh KR, Griffin SJ, van Sluijs EMF. UK Preschool-aged children's physical activity levels in childcare and at home: a cross-sectional exploration. *Int J Behav Nutr Phys Act* 2015;12:123.

26 Office for Standards in Education (Ofsted). Types of early years and childcare Ofsted inspects. 2013. http://www.ofsted.gov.uk/early-years-and-childcare (accessed 29 Nov 2014).

27 UK Government. English Indices of Deprivation 2010. https://www.gov.uk/government/statistics/english-indices-of-deprivation-2010 (accessed 4 Jan 2013).

28 Crespi C, Alfonso A, Whaley S, et al. Validity of child anthropometric measurements in the Special Supplemental Nutrition Program for Women, Infants and Children. *Pediatr Res* 2012;**71**:286–92.

29 MRC Epidemiology Unit, University of Cambridge. Simple measurements - skinfolds. DAPA Toolkit. http://dapa-toolkit.mrc.ac.uk/anthropometry/objective-methods/simple-measures-skinfolds (accessed 4 Feb 2018).

For peer review only - http://bmjopen.bmj.com/site/about/guidelines.xhtml

30 McMinn AM, van Sluijs EM, Harvey NC, et al. Validation of a maternal questionnaire on correlates of physical activity in preschool children. *Int J Behav Nutr Phys Act* 2009;6:81.

31 Ammerman AS, Ward DS, Benjamin SE, et al. An Intervention to Promote Healthy weight: Nutrition and Physical Activity Self-Assessment for Child Care (NAP SACC) theory and design. *Prev Chronic Dis* 2007;4:A67.

32 Cole TJ, Freeman J V, Preece MA. Body mass index reference curves for the UK, 1990. *Arch Dis Child* 1995;73:25–9.

33 Cole TJ, Lobstein T. Extended international (IOTF) body mass index cut-offs for thinness, overweight and obesity. *Pediatr Obes* 2012;7:284–94.

34 Freedman DS, Katzmarzyk PT, Dietz WH, et al. Relation of body mass index and skinfold thicknesses to cardiovascular disease risk factors in children : the Bogalusa Heart Study 1 – 4. *Am J Clin Nutr* 2009;90:210–6.

35 Goldstein H. *Multilevel Statistical Models*. 4th ed. Wiley-Blackwell 2010.

36 Hesketh KR, van Sluijs EMF. Features of the UK childcare environment and associations with preschooler's in-care physical activity. *Prev Med Reports* 2016;3:53–7.

37 The Office for Standards in Education Children's Services and Skills (Ofsted). Early years and childcare registration handbook. Manchester, UK: 2017. https://www.gov.uk/government/uploads/system/uploads/attachment_data/file/650103/EY_and_childcare_reg_handbook.pdf (accessed 23 Feb 2018).

38 Zahir N, Heyman MB, Wojcicki JM. No association between childcare and obesity at age 4 in low-income Latino children. *Pediatr Obes* 2012;8:E24-28.

39 Wolfenden L, Hardy LL, Wiggers J, et al. Prevalence and socio-demographic associations of overweight and obesity among children attending child-care services in

For peer review only - http://bmjopen.bmj.com/site/about/guidelines.xhtml

rural and regional Australia. *Nutr Diet* 2011;68:15–20.

40 Bower JK, Hales DP, Tate DF, et al. The Childcare Environment and Children's Physical Activity. *Am J Prev Med* 2008;34:23–9.

41 Martyniuk OJ, Vanderloo LM, Irwin JD, et al. Comparing the nutrition environment and practices of home- and centre-based child-care facilities. *Public Health Nutr* 2016;19:575–84.

42 Black L, Matvienko-Sikar K, Kearney PM. The association between childcare arrangements and risk of overweight and obesity in childhood: a systematic review. *Obes Rev* 2017;18:1170–90.

43 Gubbels JS, Kremers SPJ, Stafleu A, et al. Child-care use and the association with body mass index and overweight in children from 7 months to 2 years of age. *Int J Obes* 2010;34:1480–6.

44 Scully H, Alberdi G, Segurado R, et al. Child Care Exposure Influences Childhood Adiposity at 2 Years: Analysis from the ROLO Study. *Child Obes* 2017;13:93–101.

45 Benjamin SE, Rifas-Shiman SL, Taveras EM, et al. Early child care and adiposity at ages 1 and 3 years. *Pediatrics* 2009;124:555–62.

46 Vanderloo LM, Tucker P, Johnson AM, et al. Environmental Influences on Preschoolers' Physical Activity Levels in Various Early-Learning Facilities. *Res Q Exerc Sport* 2015;86:360–70.

47 Department for Education. Early Years Foundation Stage (EYFS). United Kingdom: 2012. http://www.education.gov.uk/aboutdfe/statutory/g00213120/eyfs-statutory-framework (accessed 19 Mar 2013).

48 Christensen P. The health-promoting family: a conceptual framework for future research. *Soc Sci Med* 2004;59:377–87.

49 Hesketh KR, Lakshman R, van Sluijs EMF. Barriers and facilitators to young

For peer review only - http://bmjopen.bmj.com/site/about/guidelines.xhtml

children's physical activity and sedentary behaviour: a systematic review and synthesis of qualitative literature. *Obes Rev* 2017; 18(9):987-1017

50 Office of National Statistics. Ethnicity and National Identity in England and Wales: 2011. London, UK: 2012. http://www.ons.gov.uk/peoplepopulationandcommunity/culturalidentity/ethnicity/articles/ethnicityandnationalidentityinenglandandwales/2012-12-11 (accessed 19 Mar 2013).

For peer review only

Supplementary Table S1: Associations between childcare attendance and environment and BMI z-score (n=196)

	Exposure Measures β [95% CI]							
	Weekly hours in care		EPAO PA score		EPAO Nutrition score		EPAO total score	
	Unadjusted	Adjusted	Unadjusted	Adjusted	Unadjusted	Adjusted	Unadjusted	Adjusted
Constant	0.51	-0.35	0.62	-0.50	0.36	-0.45	0.58	-0.39
	[0.22,0.81]	[-1.27,0.56]	[-0.52,1.77]	[-2.02,1.02]	[-0.82,1.53]	[-1.91,1.00]	[-1.11,2.26]	[-2.33,1.56]
**Child factors**								
Ethnicity (ref: white)								
White European		-0.06		-0.10		-0.10		-0.10
		[-0.56,0.44]		[-0.61,0.40]		[-0.61,0.40]		[-0.61,0.41]
Other/ mixed		0.09		0.05		0.05		0.05
		[-0.30,0.49]		[-0.35,0.45]		[-0.35,0.44]		[-0.34,0.45]
**Maternal Factors**								
Maternal BMI		0.05^{**}		0.05^{***}		0.05^{***}		0.05^{***}
		[0.02,0.08]		[0.02,0.08]		[0.02,0.08]		[0.02,0.08]
Maternal education (ref: GCSE/ A-levels)								
Degree		-0.49^{**}		-0.52^{**}		-0.52^{**}		-0.52^{**}
		[-0.83,-0.15]		[-0.86,-0.18]		[-0.86,-0.18]		[-0.86,-0.18]
Higher degree		0.04		0.01		0.01		0.01
		[-0.31,0.39]		[-0.34,0.35]		[-0.33,0.36]		[-0.33,0.35]
Maternal working hours (ref: not employed)								
<20 hours		-0.37		-0.40		-0.40		-0.40
		[-0.79,0.04]		[-0.81,0.02]		[-0.81,0.02]		[-0.81,0.02]
21-35 hours		-0.01		-0.06		-0.06		-0.05
		[-0.38,0.36]		[-0.42,0.30]		[-0.41,0.30]		[-0.41,0.31]
>35 hours		0.20		0.09		0.09		0.09
		[-0.21,0.61]		[-0.29,0.47]		[-0.29,0.47]		[-0.29,0.47]

95% confidence intervals in brackets; * $p < 0.05$, ** $p < 0.01$, *** $p < 0.001$

For peer review only

For peer review only - http://bmjopen.bmj.com/site/about/guidelines.xhtml

Supplementary Table S2: Associations between childcare attendance and environment and Waist to Height Ratio (n=188)

	Exposure Measures β [95% CI]							
	Weekly hours in care		EPAO PA score		EPAO Nutrition score		EPAO total score	
	Unadjusted	Adjusted	Unadjusted	Adjusted	Unadjusted	Adjusted	Unadjusted	Adjusted
Constant	0.48***	0.50***	0.42***	0.44***	0.51***	0.53***	0.44***	0.47***
	[0.36,0.60]	[0.36,0.65]	[0.25,0.59]	[0.24,0.64]	[0.34,0.67]	[0.35,0.71]	[0.23,0.65]	[0.24,0.71]
**Child factors**								
Sex	0.01	0.01	0.01	0.01	0.01	0.01	0.01	0.01
	[-0.02,0.03]	[-0.02,0.03]	[-0.02,0.03]	[-0.02,0.03]	[-0.02,0.03]	[-0.02,0.03]	[-0.02,0.03]	[-0.02,0.03]
Age in months	0.00	0.00	0.00	0.00	0.00	0.00	0.00	0.00
	[-0.00,0.00]	[-0.00,0.00]	[-0.00,0.00]	[-0.00,0.00]	[-0.00,0.00]	[-0.00,0.00]	[-0.00,0.00]	[-0.00,0.00]
Ethnicity (ref: white)								
White European		0.01		0.00		0.01		0.00
		[-0.04,0.06]		[-0.05,0.05]		[-0.04,0.05]		[-0.04,0.05]
Other/ mixed		0.00		-0.00		-0.00		-0.00
		[-0.04,0.04]		[-0.04,0.04]		[-0.04,0.04]		[-0.04,0.04]
**Maternal Factors**								
Maternal BMI		-0.00		-0.00		-0.00		-0.00
		[-0.00,0.00]		[-0.00,0.00]		[-0.00,0.00]		[-0.00,0.00]
Maternal education (ref: GCSE/ A-levels)								
Degree		0.00		0.00		-0.00		0.00
		[-0.03,0.03]		[-0.03,0.03]		[-0.03,0.03]		[-0.03,0.03]
Higher degree		-0.00		-0.00		-0.00		-0.00
		[-0.03,0.03]		[-0.03,0.03]		[-0.04,0.03]		[-0.04,0.03]
Maternal working hours (ref: not employed)								
<20 hours		0.01		0.00		0.00		0.00
		[-0.03,0.05]		[-0.04,0.04]		[-0.04,0.04]		[-0.04,0.04]
21-35 hours		0.01		0.01		0.01		0.01
		[-0.02,0.05]		[-0.03,0.04]		[-0.03,0.04]		[-0.03,0.04]
> 35 hours		-0.00		-0.01		-0.01		-0.01
		[-0.04,0.03]		[-0.05,0.02]		[-0.05,0.02]		[-0.05,0.02]

95% confidence intervals in brackets; * $p < 0.05$, ** $p < 0.01$, *** $p < 0.001$

For peer review only

Supplementary Table S3: Associations between childcare attendance and environment and sum of skinfolds thickness (n=144)

	Exposure Measures β [95% CI]							
	Weekly hours in care		EPAO PA score		EPAO Nutrition score		EPAO total score	
	Unadjusted	Adjusted	Unadjusted	Adjusted	Unadjusted	Adjusted	Unadjusted	Adjusted
Constant	15.12***	11.66***	15.71***	10.17*	16.38***	14.30***	15.96***	12.09*
	[9.80,20.44]	[6.03,17.30]	[8.20,23.23]	[2.07,18.27]	[9.12,23.64]	[6.81,21.78]	[6.75,25.17]	[2.43,21.76]
**Child factors**								
Sex	1.18*	1.41**	1.25*	1.53**	1.24*	1.52**	1.25*	1.53**
	[0.18,2.19]	[0.47,2.35]	[0.23,2.27]	[0.57,2.49]	[0.22,2.26]	[0.57,2.48]	[0.23,2.27]	[0.57,2.49]
Age in months	-0.02	-0.05	-0.03	-0.06	-0.03	-0.06	-0.03	-0.06
	[-0.13,0.09]	[-0.16,0.05]	[-0.14,0.08]	[-0.17,0.05]	[-0.14,0.08]	[-0.17,0.05]	[-0.14,0.08]	[-0.17,0.05]
Ethnicity (ref: white)								
White European		-0.18		-0.35		-0.20		-0.25
		[-1.94,1.58]		[-2.17,1.48]		[-2.00,1.61]		[-2.07,1.58]
Other/ mixed		-0.04		-0.01		0.08		0.10
		[-1.51,1.42]		[-1.51,1.50]		[-1.40,1.55]		[-1.39,1.59]
**Maternal Factors**								
Maternal BMI		0.27***		0.27***		0.27***		0.27***
		[0.15,0.40]		[0.15,0.40]		[0.15,0.40]		[0.14,0.40]
Maternal education (ref: GCSE/ A-levels)								
Degree		-1.61**		-1.46*		-1.49*		-1.49*
		[-2.81,-0.41]		[-2.67,-0.24]		[-2.70,-0.28]		[-2.71,-0.28]
Higher degree		-0.27		0.13		0.16		0.09
		[-1.54,1.00]		[-1.13,1.40]		[-1.11,1.44]		[-1.18,1.35]
Maternal working hours (ref: not employed)								
<20 hours		-1.03		-0.93		-0.93		-0.90
		[-2.44,0.37]		[-2.36,0.50]		[-2.36,0.50]		[-2.33,0.53]
21-35 hours		-0.30		-0.01		0.07		0.04
		[-1.64,1.04]		[-1.34,1.31]		[-1.24,1.39]		[-1.29,1.37]
>35 hours		-0.36		-0.05		-0.04		-0.05
		[-1.82,1.09]		[-1.42,1.32]		[-1.41,1.33]		[-1.42,1.33]

95% confidence intervals in brackets; * $p < 0.05$, ** $p < 0.01$, *** $p < 0.001$

For peer review only

For peer review only - http://bmjopen.bmj.com/site/about/guidelines.xhtml

STROBE 2007 (v4) Statement—Checklist of items that should be included in reports of *cross-sectional studies*

Section/Topic	Item #	Recommendation	Reported on page #
**Title and abstract**	1	(*a*) Indicate the study's design with a commonly used term in the title or the abstract	2
		(*b*) Provide in the abstract an informative and balanced summary of what was done and what was found	2
**Introduction**			
Background/rationale	2	Explain the scientific background and rationale for the investigation being reported	3-5
Objectives	3	State specific objectives, including any prespecified hypotheses	5
**Methods**			
Study design	4	Present key elements of study design early in the paper	6
Setting	5	Describe the setting, locations, and relevant dates, including periods of recruitment, exposure, follow-up, and data collection	6-7
Participants	6	(*a*) Give the eligibility criteria, and the sources and methods of selection of participants	6
Variables	7	Clearly define all outcomes, exposures, predictors, potential confounders, and effect modifiers. Give diagnostic criteria, if applicable	6-8
Data sources/ measurement	8*	For each variable of interest, give sources of data and details of methods of assessment (measurement). Describe comparability of assessment methods if there is more than one group	6-8
Bias	9	Describe any efforts to address potential sources of bias	
Study size	10	Explain how the study size was arrived at	6/ 10
Quantitative variables	11	Explain how quantitative variables were handled in the analyses. If applicable, describe which groupings were chosen and why	6--9
Statistical methods	12	(*a*) Describe all statistical methods, including those used to control for confounding	9
		(*b*) Describe any methods used to examine subgroups and interactions	9-10
		(*c*) Explain how missing data were addressed	10
		(*d*) If applicable, describe analytical methods taking account of sampling strategy	9/10
		(*e*) Describe any sensitivity analyses	9/10
**Results**			

For peer review only

Participants	13*	(a) Report numbers of individuals at each stage of study—eg numbers potentially eligible, examined for eligibility, confirmed eligible, included in the study, completing follow-up, and analysed	10
		(b) Give reasons for non-participation at each stage	10
		(c) Consider use of a flow diagram	-
Descriptive data	14*	(a) Give characteristics of study participants (eg demographic, clinical, social) and information on exposures and potential confounders	12
		10	10
Outcome data	15*	Report numbers of outcome events or summary measures	10/11
Main results	16	(*a*) Give unadjusted estimates and, if applicable, confounder-adjusted estimates and their precision (eg, 95% confidence interval). Make clear which confounders were adjusted for and why they were included	14/15
		(*b*) Report category boundaries when continuous variables were categorized	-
		(*c*) If relevant, consider translating estimates of relative risk into absolute risk for a meaningful time period	-
Other analyses	17	Report other analyses done—eg analyses of subgroups and interactions, and sensitivity analyses	14
**Discussion**			
Key results	18	Summarise key results with reference to study objectives	16
Limitations	19	Discuss limitations of the study, taking into account sources of potential bias or imprecision. Discuss both direction and magnitude of any potential bias	20/21
Interpretation	20	Give a cautious overall interpretation of results considering objectives, limitations, multiplicity of analyses, results from similar studies, and other relevant evidence	22
Generalisability	21	Discuss the generalisability (external validity) of the study results	21
**Other information**			
Funding	22	Give the source of funding and the role of the funders for the present study and, if applicable, for the original study on which the present article is based	23

*Give information separately for cases and controls in case-control studies and, if applicable, for exposed and unexposed groups in cohort and cross-sectional studies.

Note: An Explanation and Elaboration article discusses each checklist item and gives methodological background and published examples of transparent reporting. The STROBE checklist is best used in conjunction with this article (freely available on the Web sites of PLoS Medicine at http://www.plosmedicine.org/, Annals of Internal Medicine at http://www.annals.org/, and Epidemiology at http://www.epidem.com/). Information on the STROBE Initiative is available at www.strobe-statement.org.

For peer review only
